# The challenges of research data management in cardiovascular science: a DGK and DZHK position paper—executive summary

**DOI:** 10.1007/s00392-023-02303-3

**Published:** 2023-10-17

**Authors:** Sabine Steffens, Katrin Schröder, Martina Krüger, Christoph Maack, Katrin Streckfuss-Bömeke, Johannes Backs, Rolf Backofen, Bettina Baeßler, Yvan Devaux, Ralf Gilsbach, Jordi Heijman, Jochen Knaus, Rafael Kramann, Dominik Linz, Allyson L. Lister, Henrike Maatz, Lars Maegdefessel, Manuel Mayr, Benjamin Meder, Sara Y. Nussbeck, Eva A. Rog-Zielinska, Marcel H. Schulz, Albert Sickmann, Gökhan Yigit, Peter Kohl

**Affiliations:** 1 Institute for Cardiovascular Prevention (IPEK), Ludwig-Maximilians-Universität, Munich, Germany; 2 DZHK (German Centre for Cardiovascular Research), Partner Site Munich Heart Alliance, Munich, Germany; 3 Institute for Cardiovascular Physiology, Goethe University, Frankfurt Am Main, Germany; 4 DZHK (German Centre for Cardiovascular Research), Partner Site RheinMain, Frankfurt, Germany; 5 Institute of Cardiovascular Physiology, University Hospital Düsseldorf, Düsseldorf, Germany; 6 Cardiovascular Research Institute Düsseldorf (CARID), Düsseldorf, Germany; 7 Comprehensive Heart Failure Center (CHFC), University Clinic Würzburg, Würzburg, Germany; 8 Medical Clinic 1, University Clinic Würzburg, Würzburg, Germany; 9 Clinic for Cardiology and Pneumology, Georg-August University Göttingen, Göttingen, Germany; 10 DZHK (German Center for Cardiovascular Research), Partner Site Göttingen, Göttingen, Germany; 11 Institute of Pharmacology and Toxicology, University of Würzburg, Würzburg, Germany; 12 Institute of Experimental Cardiology, University Hospital Heidelberg, Heidelberg, Germany; 13 DZHK (German Center for Cardiovascular Research), Partner Site Heidelberg/Mannheim, Heidelberg, Germany; 14 Faculty of Medicine, Institute for Experimental and Clinical Pharmacology and Toxicology, Albert-Ludwigs-University, Freiburg, Germany; 15 Department of Diagnostic and Interventional Radiology, University Hospital Würzburg, Würzburg, Germany; 16 Cardiovascular Research Unit, Department of Precision Health, Luxembourg Institute of Health, Strassen, Luxembourg; 17 Department of Cardiology, CARIM School for Cardiovascular Diseases, Maastricht University, Maastricht, The Netherlands; 18 Institute of Medical Biometry and Statistics, Faculty of Medicine and Medical Center, University of Freiburg, Freiburg, Germany; 19 Institute of Experimental Medicine and Systems Biology, RWTH Aachen Medical Faculty, Aachen, Germany; 20 Department of Nephrology and Clinical Immunology, RWTH Aachen Medical Faculty, Aachen, Germany; 21 Department of Internal Medicine, Nephrology and Transplantation, Erasmus MC, Rotterdam, The Netherlands; 22 Department of Cardiology, Maastricht University Medical Centre and Cardiovascular Research Institute Maastricht, Maastricht, The Netherlands; 23 Department of Biomedical Sciences, Faculty of Health and Medical Sciences, University of Copenhagen, Copenhagen, Denmark; 24 Oxford E-Research Centre (OeRC), Department of Engineering Science, University of Oxford, Oxford, UK; 25 Max Delbrück Center for Molecular Medicine in the Helmholtz Association, Berlin, Germany; 26 DZHK (German Centre for Cardiovascular Research), Partner Site Berlin, Berlin, Germany; 27 Department for Vascular and Endovascular Surgery, Klinikum Rechts Der Isar, Technical University Munich, Munich, Germany; 28 Department of Medicine, Karolinska Institute, Stockholm, Sweden; 29 School of Cardiovascular Medicine and Sciences, King’s College London British Heart Foundation Centre, London, UK; 30 Division of Cardiology, Department of Internal Medicine II, Medical University of Vienna, Vienna, Austria; 31 Department of Internal Medicine III (Cardiology, Angiology, and Pneumology), University Hospital Heidelberg, Heidelberg, Germany; 32 Department of Medical Informatics, University Medical Center Göttingen (UMG), Göttingen, Germany; 33 Central Biobank UMG, UMG, Göttingen, Germany; 34 Institute for Experimental Cardiovascular Medicine, University Heart Center Freiburg-Bad Krozingen, University of Freiburg, Freiburg, Germany; 35 Faculty of Medicine, University of Freiburg, Freiburg, Germany; 36 Institute of Cardiovascular Regeneration, Goethe University, Frankfurt, Germany; 37 Leibniz-Institut Für Analytische Wissenschaften, ISAS, E.V., Dortmund, Germany; 38 Department of Chemistry, College of Physical Sciences, University of Aberdeen, Aberdeen, UK; 39 Institute for Virology, University Hospital Essen, University of Duisburg-Essen, Essen, Germany; 40 Institute of Human Genetics, University Medical Center Göttingen, Göttingen, Germany; 41 German Center of Cardiovascular Research (DZHK), Partner Site Göttingen, Göttingen, Germany; 42 CIBSS Centre for Integrative Biological Signalling Studies, University of Freiburg, Freiburg, Germany

**Keywords:** Research data management, FAIR data, Metadata, Cardiac research, Cardiology

## Abstract

The sharing and documentation of cardiovascular research data are essential for efficient use and reuse of data, thereby aiding scientific transparency, accelerating the progress of cardiovascular research and healthcare, and contributing to the reproducibility of research results. However, challenges remain. This position paper, written on behalf of and approved by the German Cardiac Society and German Centre for Cardiovascular Research, summarizes our current understanding of the challenges in cardiovascular research data management (RDM). These challenges include lack of time, awareness, incentives, and funding for implementing effective RDM; lack of standardization in RDM processes; a need to better identify meaningful and actionable data among the increasing volume and complexity of data being acquired; and a lack of understanding of the legal aspects of data sharing. While several tools exist to increase the degree to which data are findable, accessible, interoperable, and reusable (FAIR), more work is needed to lower the threshold for effective RDM not just in cardiovascular research but in all biomedical research, with data sharing and reuse being factored in at every stage of the scientific process. A culture of open science with FAIR research data should be fostered through education and training of early-career and established research professionals. Ultimately, FAIR RDM requires permanent, long-term effort at all levels. If outcomes can be shown to be superior and to promote better (and better value) science, modern RDM will make a positive difference to cardiovascular science and practice. The full position paper is available in the supplementary materials.

**Supplementary Information:**

The online version contains supplementary material available at 10.1007/s00392-023-02303-3.

## Introduction

Modern cardiovascular research has been associated with a rapid increase in the volume of data obtained by cardiovascular researchers as data are collected at ever finer levels of structural and functional complexity. At the same time, governments, funders, and journals have begun to encourage or require the sharing of research data in a findable, accessible, interoperable, and reusable (‘FAIR’) way [1–5].

However, there are significant barriers to effective and responsible research data management (RDM) in cardiovascular science that must be overcome. First, there is a lack of standardization in how data and metadata are collected, processed, and shared. Second, researchers often lack sufficient time, funding, or incentives to share their data. Third, the volume and complexity of data being collected makes the identification of meaningful and actionable data increasingly difficult. Finally, there are complex ethical and legal aspects of sharing data that researchers should understand, for example when dealing with sensitive data or sharing data across the borders of the European Union. Addressing these challenges will make it easier for scientists to use and understand their own data and the data of others.

The 3rd Joint German Cardiac Society (DGK) and German Centre for Cardiovascular Research (DZHK) Translational Workshop was held in Bonn, Germany in September 2022 to discuss the challenges and potential solutions associated with RDM in cardiovascular research; the topics, opinions, and findings discussed during the workshop are presented here. This position paper executive summary, written on behalf of and endorsed by the DGK and DZHK, identifies and describes challenges that scientists and clinicians currently face when collecting, using, and reusing data in the field of cardiovascular research and beyond. It then provides recommendations for improvements in RDM practices, informed by standardization efforts and guidelines from related domains. The full position paper can be found in the supplementary materials.

## Data sharing and metadata

**Fig. 1:**
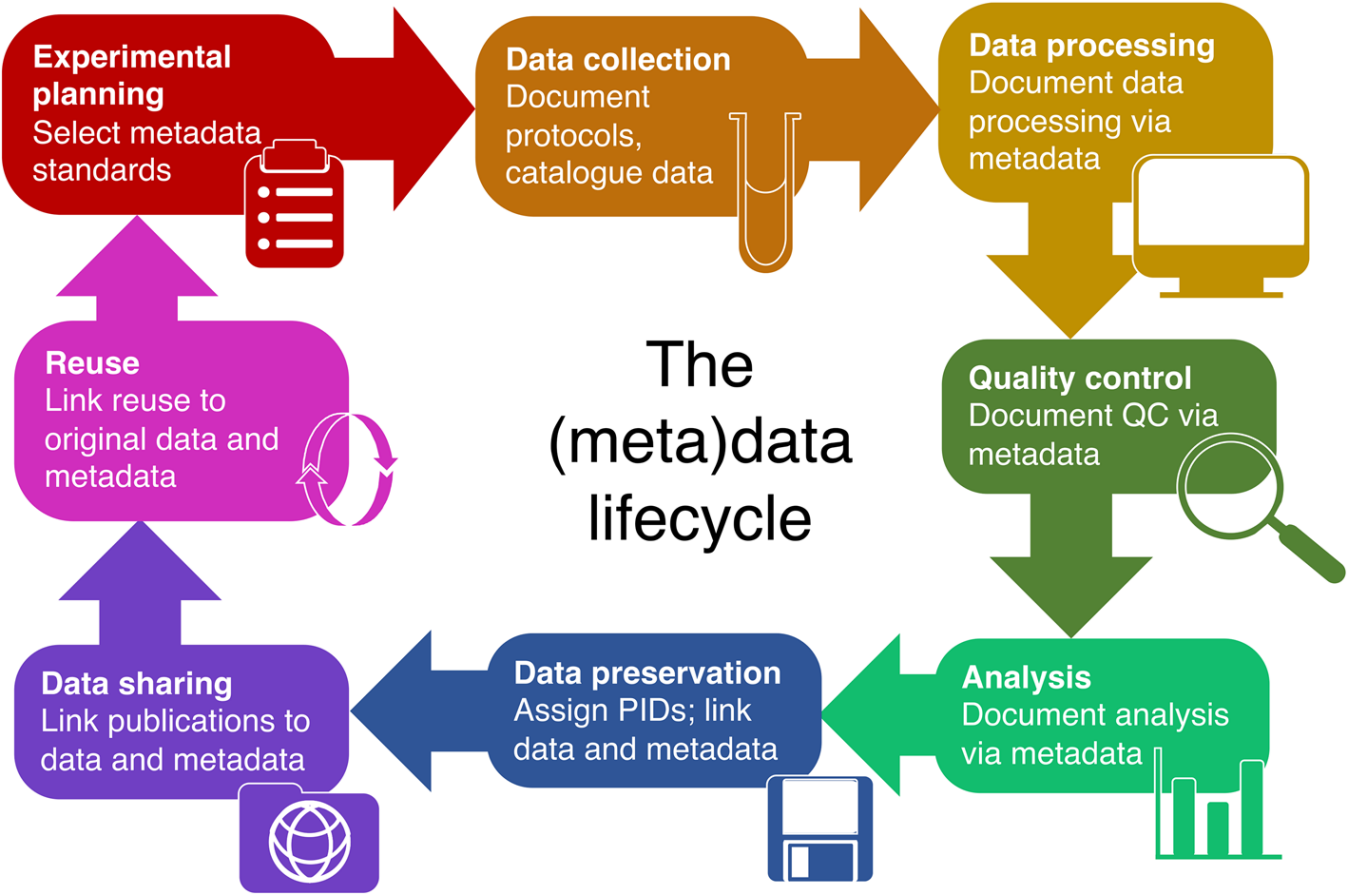
The data and metadata lifecycle. *PID* permanent identifier, *QC* quality control

Effective RDM involves every step of the data lifecycle (Fig. 1) [6]. When planning a study, we recommend that researchers formulate a data management plan that considers how data and metadata will be collected, stored, annotated, analyzed, and shared [7–10]. Sharing data increases its impact [11] and allows researchers, peer-reviewers, and journals to understand exactly how work was carried out and accurately assess its validity even long after the conclusion of a study [10].

Metadata (‘data about data’) provide essential information on the context, quality, structure, and condition of data [10, 12, 13]. Metadata and data should be linked together via unique and persistent identifiers and using standards such as FAIR digital objects (https://fairdo.org/) and the Research Object Crate (RO-Crate; https://www.researchobject.org) format [14], which aim to provide a mechanism to not only link data and metadata but also the associated analysis workflows, software, protocols, publications, presentations, and licensing information. Although metadata documentation is ultimately the responsibility of the investigators who collect the data, researchers may lack the time or expertise to generate good-quality metadata [13]. Training and education are needed, and the adoption or adaptation of community standards, such as the Recommended Metadata for Biological Images (REMBI) [15] and Investigation Study Assay (ISA) framework (https://isa-tools.org/) [16], is recommended to ensure consistent use of metadata. Where community standards do not yet exist, research teams are encouraged to self-organize and create common reporting formats for data and metadata, and to document them publicly [17]. Research teams may also enlist the services of specialist ‘data stewards’ to assist with data management, including metadata documentation; these roles require funds that are usually not available, however.

## The FAIR principles

The FAIR principles for scientific data management and stewardship provide comprehensive and practical guidelines for ensuring data and metadata are FAIR (http://www.go-fair.org/fair-principles) [18]. Funders, journals, and policymakers are increasingly requiring the implementation of the FAIR principles for all research data and other related digital objects. While the FAIR principles provide a framework, it remains the responsibility of researchers to decide how they will ensure their data and metadata are FAIR. Fortunately, there are materials that provide guidance on implementing the FAIR principles at the project, group, and institutional levels (Table 1). However, to ensure widespread adoption of the FAIR principles, the threshold for implementation needs to be reduced substantially, such as with simple-to-use tools for easy data deposition and access via dedicated repositories.

**Table 1:** Examples of standards and tools that researchers may use to make their data FAIR

Example	Description
Research Data Alliance https://www.rd-alliance.org/	A global, community-driven initiative to build social and technical infrastructure for open sharing and reuse of data, with several working groups in a number of disciplines
FAIRsharing http://www.fairsharing.org	A searchable, interconnected registry of data standards, databases, and data policies across many research areas, allowing researchers to discover relevant repositories that meet their requirements [16]
The FAIR Cookbook https://faircookbook.elixir-europe.org	A collection of practical ‘recipes’ that provide guidance on the operational steps of FAIR data management, from creating unique, persistent identifiers to declaring data’s permitted uses [32]
FAIRassist.org https://fairassist.org	A repository aiming to offer personalized guidance to discover FAIR standards and other resources such as the Data Stewardship Wizard
FAIR Data Self Assessment Tool https://ardc.edu.au/resource/fair-data-self-assessment-tool/	Self-assessment tool from the Australian Research Data Commons that allows users to assess how FAIR their research dataset is by answering simple questions
ELIXIR Research Data Management Kit (RDMKit) https://rdmkit.elixir-europe.org/	Provides a set of best practices and guidelines for FAIR RDM across several life science domains, and journal research data policies [33]
OpenAIRE https://www.openaire.eu	Provides resources for researchers for the management and interoperability of data
Minimum Information about a Cardiac Electrophysiology Experiment (MICEE) [31]	An example of minimum reporting standards for recording, annotating, and reporting data from cardiac electrophysiology experiments
‍FAIRsFAIR Data Policy Checklist https://www.fairsfair.eu/sites/default/files/FsF_Structured_Policy_Descriptions_17022022.pptx.pdf	The FAIRsFAIR FAIR Data Policy Checklist and related structured policy description template provide support for the creation of structured policy documents at the project, institutional, and community level, helping policymakers to assess whether elements of their data policies are FAIR-enabling

## Data sharing and FAIR research to reduce the costs of scientific research

The financial costs of non-FAIR data can be quantified: a study by the European Commission estimated that non-FAIR research data costs €10.2 billion per year in Europe, with an additional estimated €16 billion impact on innovation; it is worth mentioning that these figures do not include the non-quantifiable benefits of making data FAIR [19]. Making data FAIR will increase the value of the data obtained, potentially accelerate progress in the improvement of therapeutics and diagnostics, and maximize the return on investment for funders.

## Research data management challenges in cardiovascular science

### Sharing data via data repositories and encouraging data sharing

Data repositories are centralized storage spaces where datasets can be deposited for access and reuse by other users (although authorisation and authentication may be required). There are several thousand data repositories, ranging from generalist repositories to specialist repositories for specific kinds of data. At minimum, repositories should automatically provide a globally unique and persistent identifier to every element of each dataset [20]. Repositories should also require deposition of sufficient metadata to allow other users to understand, process, and compare the data in a meaningful way. Services such as re3data (https://www.re3data.org) or FAIRsharing (http://www.fairsharing.org, [16]) provide a means to discover relevant repositories that meet FAIR requirements.

If data repositories are to realize their potential, it will be necessary to further encourage contribution as outlined below. Publications that are linked to the underlying research data are already cited more often [11]. Researchers and journals should ensure that data are credited or cited wherever they are used or reused, for example by crediting the investigators who collected the original data and citing the original dataset [21–23]. Researchers who share well-annotated datasets via repositories should be recognized and rewarded by funding bodies and universities in a suitable manner [22, 23]. Citing data sources would allow some academic recognition and reward for data sharing, and help researchers satisfy funding obligations to share their data [24]. Researchers should work with their community to define expectations for the management, sharing, and reuse of data and associated metadata, for example by joining an already existing initiative such as one of the many working and interest groups within the Research Data Alliance (https://rd-alliance.org/).

### Managing data heterogeneity: standardization and harmonization

Sharing and combining datasets can be challenging due to the heterogeneity of the data involved, particularly if data are obtained using team-specific protocols and with limited standardization across laboratories. Greater standardization of terminology and better adherence to existing standards is needed across cardiovascular science. Standardized collection, processing, quality assessment, and analysis pipelines are also needed to ensure interoperability and comparability of data. In evolving fields, there may be a need to develop and adopt community-wide standards for the collection and preservation of data and metadata ‘on-the-job’. Community reporting guidelines (or minimum information standards) that describe how to report everything from sample quality to the data processing protocols used can facilitate data sharing, streamline workflows, and allow for the long-term preservation of and access to information [17, 25, 26]. Where reporting standards do not exist, research communities can self-organize and create community-centric reporting formats for data and metadata [17]. Large-scale collaborative initiatives like the National Sleep Research Resource (http://www.sleepdata.org) and the UK Biobank (https://www.ukbiobank.ac.uk) have shown it is possible for researchers to organize and collaborate on the collection and sharing of large volumes of health data for their mutual benefit, despite the challenges. Although it is important to recognize that there is unlikely to be a ‘one-size-fits-all’ solution, these initiatives may provide a model for similar efforts in cardiovascular science.

### Identifying meaningful, actionable data

Given the volume of data that can be collected with modern high-throughput techniques and novel technologies like mobile health devices, it is important that researchers focus on data that are meaningful (e.g., relevant to the disease being studied) and actionable (e.g., useful for answering a specific research question or to inform a specific treatment decision). Better guidance from manufacturers is needed to ensure researchers and clinicians can effectively use the most appropriate available technologies, better identify useful meaningful and actionable data, and improve treatment decision-making and risk assessment. Collaborations between scientific researchers, healthcare providers, manufacturers, software developers, and insurance companies may provide an opportunity to influence and guide the development of new technology to improve the quality and utility of data collected.

### Managing sensitive data

Sharing sensitive data is rightfully strictly regulated, but levels of regulation differ internationally [27, 28]. Education is needed to ensure that researchers understand when and how data may be shared, what researchers need to do to ensure that they are in compliance with applicable laws (e.g., GDPR), and what technology is available for secure data sharing.

In research involving patients, consent must be managed and documented appropriately. Patients often support sharing of their data if it will improve diagnostic and therapeutic options [29, 30], but lack of information on the exact parameters of consent may prevent reuse of data where consent exists but is not easily traceable. Digitization and automation of (remote) patient consent is increasing and may help to improve access to samples and data as details of patient consent can be more efficiently traced.

## Who is responsible for RDM in cardiovascular science?

Researchers are ultimately responsible for ensuring that research data are suitably managed and shared according to the FAIR principles, including ensuring that data are adequately documented with metadata and made available for reuse as appropriate.

Journals and publishers should require authors to include links to all relevant raw or processed data, metadata, and other relevant materials in their submissions when publicly available. Where data are not publicly available, data sharing statements should indicate how the data can be accessed or requested, and authors or organizations should be expected to make data available upon request. Journals should also take greater responsibility for confirming that submitted materials include working links to the raw data, metadata, and other relevant materials, ideally via a persistent identifier (although the ultimate responsibility will continue to lie with authors to provide working links at submission).

Universities, funders, and government bodies should recognize and reward the collection and sharing of data in the same way that they recognize and reward publication activity. They should also support effective RDM via education and training programs, by defining and implementing data sharing policies, by employing data stewards, and by providing sufficient long-term funds for data storage and sharing. They should also establish data management training modules at the graduate level, with more advanced training at post-doctoral levels. In Germany, there is a chance to accelerate progress in the education of physicians in terms of the collection, use, quality assessment, and analysis of data via updates to the *Approbationsordnung.* Institutions, publishers, and state and national government bodies should commit to improving and future-proofing digital infrastructures for data storage and sharing, including funding for relevant personnel. Researchers, institutions, and journals should work together to develop low-threshold tools for data and metadata sharing during data acquisition (electronic records), processing (automated metadata annotation), and publication (low-level access to key data, such as contained in figures, via a ‘data container’). Coordination of RDM practices remains a challenge; currently, the German Research Foundation encourages RDM policy development by each ‘network grant’. Whether this is the most effective way forward remains to be seen, as parallel work, at times even within one university or faculty, would seem counterproductive. While the legal hurdles that need to be overcome when sharing data between, for example, EU- and non-EU-based research teams may be alleviated with technical solutions, a definitive solution will require the involvement of national funding bodies and governmental entities.

Finally, the cardiovascular research community should work to make sharing raw data and metadata the norm at all levels via the creation or adoption of cardiovascular reporting guidelines (an example of a well-intentioned and broadly endorsed—yet under-utilized—reporting guideline is MICEE [31]). As much cardiovascular research data and accompanying metadata should be made available via public repositories as possible to ensure the long-term and sustainable storage and reuse of data.

## Conclusion

Data reuse should be factored in at every stage of scientific research, and researchers should foster a culture of open, FAIR science, through sharing good-quality, well-annotated data and metadata in repositories, defining and following agreed-upon standards, crediting and linking to the data of others, and publishing negative results. Community-driven standardization and harmonization at all stages of the data lifecycle is needed to reduce the heterogeneity of data and ensure good data quality. However, it is important to recognize that there is unlikely to be a one-size-fits-all solution for effective RDM in cardiovascular science, and the development, adoption, and application of RDM practices will require careful consideration at all levels and in all areas of cardiovascular research and should be part of the new *Ärztliche Approbationsordnung*. Standards should be considered living documents that need to be regularly adapted to new technologies or methods. Education, training, and funding are essential for widespread and enduring adoption of effective RDM.

It is not enough to simply recognize the importance of responsible and effective RDM: it must be put into practice. The authors encourage their professional societies and research organizations (including the DGK and DZHK), as well as funding and regulatory bodies, to spearhead a number of initiatives, including: (1) supporting initiatives and/or lobbying national funding bodies to aid a more concerted effort to develop relevant RDM processes and tools and FAIR data sharing approaches across the life sciences, including the development of and adherence to minimum reporting guidelines; (2) developing a generic (PDF- or HTML-compatible), pragmatic (focusing on data used to create figures in peer-reviewed publications), low-threshold (data container) tool to make a first but definitive step to data sharing that is independent of the research subject, methods used, and level of investigation involved; and (3) pushing for generalized ‘point-of-entry’ consenting of patients for the use of their data and any biological materials acquired in the process of diagnostic or therapeutic interventions that would otherwise be discarded, and probing the ethical acceptability of discarding healthy human donor tissue (the biological reference) that may not be used clinically (which must remain the primary aim of all donor organ utilization).

### Supplementary Information

Below is the link to the electronic supplementary material.

Supplementary file1 (DOCX 224 kb)

## Data Availability

Not applicable.
