# Supplementary material for: The challenges of research data management in cardiovascular science: a DGK and DZHK position paper—executive summary
Source: Clin Res Cardiol. 2023 Oct 17;113(5):672–9. doi: 10.1007/s00392-023-02303-3 (PMC11026239; doi:10.1007/s00392-023-02303-3)
Supplement: Supplementary file 1 — Supplementary file1 (DOCX 224 kb) [file 392_2023_2303_MOESM1_ESM.docx]

# Supplementary Material

The challenges of research data management in cardiovascular science: a DGK and DZHK position paper

Sabine Steffens^1,2^ • Katrin Schröder^3,4^ • Martina Krüger^5,6^ • Christoph Maack^7,8^ • Katrin Streckfuss-Bömeke^9,10,11^ • Johannes Backs^12,13^ • Rolf Backofen^14^ • Bettina Baeßler^15^ • Yvan Devaux^16^ • Ralf Gilsbach^12,13^ • Jordi Heijman^17^ • Jochen Knaus^18^ • Rafael Kramann^19,20,21^ • Dominik Linz^22,23^ • Allyson L. Lister^24^ • Henrike Maatz^25,26^ • Lars Maegdefessel^27,28,2^ • Manuel Mayr^29,30^ • Benjamin Meder^13,31^ • Sara Y. Nussbeck^32,33^ • Eva A. Rog-Zielinska^34,35^ • Marcel H. Schulz^36,4^ • Albert Sickmann^37,38,39^ • Gökhan Yigit^40,41^ • Peter Kohl^34,35,42^

Sabine Steffens, Katrin Schröder, Matina Krüger, Christoph Maack, Katrin Streckfuss-Bömeke, and Peter Kohl are members of the Commission for Experimental Cardiology of the DGK and the Workshop Organising Committee.

Sabine Steffens and Katrin Schröder contributed equally to this work.

Correspondence to Peter Kohl (peter.kohl@uniklinik-freiburg.de)

^1^Institute for Cardiovascular Prevention (IPEK), Ludwig-Maximilians-Universität, Munich. ^2^DZHK (German Centre for Cardiovascular Research), partner site Munich Heart Alliance, Munich, Germany. ^3^Institute for Cardiovascular Physiology, Goethe University, Frankfurt am Main, Germany. ^4^DZHK (German Centre for Cardiovascular Research), Partner site RheinMain, Frankfurt, Germany. ^5^Institute of Cardiovascular Physiology, University Hospital Düsseldorf, Düsseldorf, Germany. ^6^Cardiovascular Research Institute Düsseldorf (CARID), Düsseldorf, Germany. ^7^Comprehensive Heart Failure Center (CHFC), University Clinic Würzburg, Würzburg, Germany. ^8^Medical Clinic 1, University Clinic Würzburg, Würzburg, Germany. ^9^Clinic for Cardiology and Pneumology, Georg-August University Göttingen, Göttingen, Germany. ^10^DZHK (German Center for Cardiovascular Research), partner site Göttingen, Göttingen, Germany. ^11^Institute of Pharmacology and Toxicology, University of Würzburg, Würzburg, Germany. ^12^Institute of Experimental Cardiology, University Hospital Heidelberg, Heidelberg, Germany. ^13^DZHK (German Center for Cardiovascular Research), Partner Site Heidelberg/Mannheim, Heidelberg, Germany. ^14^Institute for Experimental and Clinical Pharmacology and Toxicology, Faculty of Medicine, Albert-Ludwigs-University, Freiburg, Germany. ^15^Department of Diagnostic and Interventional Radiology, University Hospital Würzburg, Würzburg, Germany. ^16^Cardiovascular Research Unit, Department of Precision Health, Luxembourg Institute of Health, Strassen, Luxembourg. ^17^Department of Cardiology, CARIM School for Cardiovascular Diseases, Maastricht University, Maastricht, The Netherlands. ^18^Institute of Medical Biometry and Statistics, Faculty of Medicine and Medical Center – University of Freiburg, Germany. ^19^Institute of Experimental Medicine and Systems Biology, RWTH Aachen Medical Faculty, Aachen Germany. ^20^Department of Nephrology and Clinical Immunology, RWTH Aachen Medical Faculty, Aachen Germany. ^21^Department of Internal Medicine, Nephrology and Transplantation, Erasmus MC, Rotterdam, The Netherlands. ^22^Department of Cardiology, Maastricht University Medical Centre and Cardiovascular Research Institute Maastricht, Maastricht, the Netherlands. ^23^Department of Biomedical Sciences, Faculty of Health and Medical Sciences, University of Copenhagen, Copenhagen, Denmark. ^24^Oxford e-Research Centre (OeRC), Department of Engineering Science, University of Oxford, Oxford, UK. ^25^Max Delbrück Center for Molecular Medicine in the Helmholtz Association, Berlin, Germany. ^26^DZHK (German Centre for Cardiovascular Research), Partner Site Berlin, Berlin, Germany. ^27^Department for Vascular and Endovascular Surgery, Klinikum rechts der Isar, Technical University Munich; Munich, Germany. ^28^Department of Medicine, Karolinska Institute; Stockholm, Sweden. ^29^King's College London British Heart Foundation Centre, School of Cardiovascular Medicine and Sciences, London, UK. ^30^Department of Internal Medicine II, Division of Cardiology, Medical University of Vienna, Austria. ^31^Department of Internal Medicine III (Cardiology, Angiology, and Pneumology), University Hospital Heidelberg, Heidelberg, Germany. ^32^Department of Medical Informatics, University Medical Center Göttingen (UMG), Göttingen, Germany. ^33^Central Biobank UMG, UMG, Göttingen, Germany. ^34^Institute for Experimental Cardiovascular Medicine, University Heart Center Freiburg - Bad Krozingen, University of Freiburg, Freiburg, Germany. ^35^Faculty of Medicine, University of Freiburg, Freiburg, Germany. ^36^Institute of Cardiovascular Regeneration, Goethe University, Frankfurt, Germany. ^37^Leibniz-Institut Für Analytische Wissenschaften - ISAS - E.V., Dortmund, Germany. ^38^Department of Chemistry, College of Physical Sciences, University of Aberdeen, Aberdeen, UK. ^39^Institute for Virology, University Hospital Essen, University of Duisburg-Essen, Essen, Germany. ^40^Institute of Human Genetics, University Medical Center Göttingen, Göttingen, Germany. ^41^German Center of Cardiovascular Research (DZHK), Partner Site Göttingen, Göttingen, Germany. ^42^CIBSS Centre for Integrative Biological Signalling Studies, University of Freiburg, Freiburg, Germany.

# Abstract

The sharing and documentation of cardiovascular research data are essential for efficient use and reuse of data, thereby aiding scientific transparency, accelerating the progress of cardiovascular research and healthcare, and contributing to the reproducibility of research results. However, challenges remain. This position paper, written on behalf of and approved by the German Cardiac Society (DGK) and German Centre for Cardiovascular Research (DZHK), summarises our current understanding of the challenges in cardiovascular research data management (RDM). These challenges include a lack of time, awareness, incentives, and funding for implementing effective RDM practice; a lack of standardisation in RDM processes; a need to better identify meaningful and actionable data among the increasing volume and complexity of data being acquired; and a lack of understanding of the legal aspects of data sharing. While several tools exist to increase the degree to which data are findable, accessible, interoperable, and reusable (‘FAIR’), more work is needed to lower the threshold for effective RDM not just in cardiovascular research but in all biomedical research, with data sharing and reuse being factored in at every stage of the scientific process. A culture of open science with FAIR research data should be fostered through education and training of early-career and established research professionals. Ultimately, FAIR RDM requires permanent, long-term effort at all levels. If outcomes can be shown to be superior and to promote better (and better value) science, modern RDM will make a positive difference to cardiovascular science and practice.

# Introduction

The increasing use of –omics (multi-omics) and other high-throughput techniques, (semi-) continuous monitoring of vital parameters, and advanced high-resolution, multidimensional, and multimodal imaging has led to a rapid increase in the volume of data obtained by cardiovascular researchers. Data are collected at ever finer levels of structural and functional complexity, in an approach sometimes referred to as ‘biological reduction’ [1]. At the same time, unprecedented volumes of pre-clinical and clinical data, including so-called ‘big data’ from electronic medical records and mobile health (mHealth) devices, are now available, facilitating the study of structurally and functionally complex systems up to the societal level, in an approach sometimes referred to as ‘biological integration’. Meanwhile, governments, funders, and journals have begun to adopt policies that encourage or even require sharing of research data in a findable, accessible, interoperable, and reusable (‘FAIR’) way [2-6].

However, there are significant barriers to effective and responsible research data management (RDM) in cardiovascular science that must be overcome. First, there is a lack of standardisation in how data and metadata are collected, processed, and shared. Second, researchers often lack sufficient time, funding, or incentives to share their data. Third, the volume and complexity of data being collected makes the identification of meaningful and actionable data increasingly difficult. Finally, there are complex ethical and legal aspects of sharing data that researchers should understand, for example when dealing with sensitive data or sharing data across the borders of the European Union (EU). Aside from hindering scientific progress, these challenges also represent societal, economic, and ethical problems, as they cost valuable time and resources that could potentially delay the development of new preventative, diagnostic, and therapeutic techniques [7]. Addressing these challenges will make it easier for researchers to use and understand their own data and the data of others.

The 3^rd^ Joint German Cardiac Society (DGK) and German Centre for Cardiovascular Research (DZHK) Translational Workshop was held in Bonn, Germany, in September 2022 to discuss the challenges and potential solutions associated with RDM in cardiovascular research; the topics, opinions, and findings discussed during the workshop are presented here. This position paper, written on behalf of and endorsed by the DGK and DZHK, identifies and describes challenges that scientists and clinicians currently face when collecting, using, and reusing data in the field of cardiovascular research and beyond. It then provides recommendations for improvements in RDM practices, informed by standardisation efforts and guidelines from related domains.

# Data and metadata

## Data and the data lifecycle

Scientific data are the product of processes that may be grouped together in a ‘data lifecycle’ (Figure 1) [8], in which data from one study are used in the planning or execution of subsequent studies. Effective RDM involves every step of the data lifecycle.

**The data lifecycle begins with data stewardship – even before data collection:** Planning the long-term and sustainable care of research data (‘data stewardship’) should ideally happen before data are collected, not after. Although data management is generally well-regulated in clinical trials, this is not necessarily the case in most basic biomedical research. When planning a study, we recommend that researchers formulate a data management plan that considers how data and metadata will be collected, stored, annotated, and analysed, and how and where the research artefacts, including the data and metadata, will be shared [9-12].

**The data lifecycle does not end at publication:** Researchers should also recognise that the data lifecycle does not end at publication: the value of data is determined not only by the utility of the data for the initial investigation that motivated their collection, but also by how they are shared and reused. This can have direct benefits for researchers: publications that allow readers to access the data (e.g., by providing a link to the data in a repository) are cited more often, and therefore may be expected to have greater scientific impact [13].

**Access to data and reproducible analysis is essential for transparency:** There is an ongoing reproducibility crisis in science [14-16]. In many cases, the inability to reproduce results may be due to a lack of access to the original raw data, metadata, or other information necessary to replicate experiments. This may mean valid findings cannot be replicated, casting them into unnecessary doubt. Improved documentation and sharing of data and its processing will allow researchers, peer-reviewers, and journals to understand exactly how work was carried out, and to accurately assess the validity of published data even long after the conclusion of a study [12]. In addition, negative findings or studies documenting the inability to reproduce previous work should be made available on open-access preprint servers (such as <https://www.bioRxiv.org> or <https://www.medRxiv.org>) if they cannot be published as peer-reviewed articles. Recent initiatives by journals to address this problem, such as the open call by the *Journal of Molecular and Cellular Cardiology Plus* for papers that address scientific reproducibility and the reporting of negative results, are encouraging.

## Metadata

To use data effectively, users need to be able to understand the nature and limitations of the data. This information can be communicated using metadata that documents everything from methodological information (describing how data were obtained, processed, and analysed) to biological information (cell types, model strains, exact interventions applied, and auxiliary parameters such as environmental variables, etc.) [17].

**What are metadata?** Metadata are ‘data about data’, and provide important information on the context, quality, structure, and condition of data [12,18]. There are many types of metadata, including descriptive (e.g., species, animal protocols, sample, environmental parameters), administrative (e.g., who collected the data and when), and structural metadata (e.g., how data relate to other data in the dataset). Metadata – especially adequate annotations using recognised metadata standards – are important for ensuring that research data are FAIR [18,19].

**Who creates metadata?** Metadata documentation is ultimately the responsibility of the investigators who collect the data, but researchers may lack the time or expertise to generate good-quality metadata [18]. Some devices that generate high-throughput or high-density data (e.g., imaging systems) store relevant metadata automatically; these resources are often directly linked to acquired data, making documentation much easier. In all cases, however, metadata documentation requires expertise and care and should be carried out with access by others in mind. Protocols, for example, should be structured, use standardised terms instead of user- or lab-specific abbreviations and jargon, and they should clearly specify the equipment, materials, and software used. Analysis should also be reproducible, and metadata should document all software and database versions used, as well as the specific parameters [20]. This can be done automatically using advanced workflow management systems such as Galaxy [21].

Research teams may enlist the services of specialist ‘data stewards’ to assist with data management, including metadata documentation; these roles require funds that are usually not available, however. To maintain bioinformatics tools for sustainable and qualitative analysis of large datasets, the German government established de.NBI, a national bioinformatics infrastructure to provide first-class tools and services, and relevant training for life science and biomedicine (<https://www.denbi.de/>), enabling researchers to generate good-quality metadata.

**Metadata and data should be linked:** Metadata and the data they describe are usually separate entities; it is therefore important that metadata include the corresponding dataset’s unique and persistent identifiers (and vice versa) so that both can be linked. Researchers may wish to use standards such as FAIR digital objects (<https://fairdo.org/>) and the Research Object Crate (RO-Crate; [https://www.researchobject.org](https://www.researchobject.org/))[22] format, which aim to provide a mechanism to not only link data and metadata but also the associated analysis workflows, software, protocols, publications, presentations, and licensing information.

**Researchers should adhere to metadata standards:** Metadata standards are documents that establish how data should be structured and understood, by defining and describing the common elements that make up the metadata. The Dublin Core, for example, is a set of 15 metadata elements to help identify and organise digital resources, including the title, creator, format, and description of the resource (<https://www.dublincore.org>). Worryingly, however, many academic researchers may not use a specific metadata standard, which may severely limit the discoverability and reusability of the data and suggests that researchers need better support, training, and education concerning metadata creation [8].

**Metadata should be made available:** An analysis of thousands of single-cell RNA sequencing publications and datasets found that less than a quarter of studies provide cell-level metadata with identified cell types, even though such information is necessary for replicating prior work or reanalysis [19]. Researchers should ensure that metadata are made available along with the accompanying data.

**How can metadata documentation be improved?** Training and education may be helpful, including offerings by groups such as the International Science Council’s Committee on Data (CODATA; <https://codata.org/>). The adoption or adaptation of community standards, such as the Recommended Metadata for Biological Images (REMBI) [23] and Investigation Study Assay (ISA) framework (<https://isa-tools.org/>)[24] is recommended to ensure consistent use of metadata. ISA for example has been successfully used in conjunction with domain-specific annotation requirements such as those within the open cell migration community [25]. Similarly, minimum reporting standards have been developed for many areas of biomedical research, and (if adhered to) form an invaluable resource for FAIR RDM. Where community standards do not yet exist, research teams are encouraged to self-organise and create common reporting formats for data and metadata, and to document them publicly [26].

# The FAIR Principles

The FAIR principles for scientific data management and stewardship were defined in 2016 by a group of scientists and private organisations with an interest in overcoming the barriers to data discoverability and reuse [27]. The principles provide comprehensive and practical guidelines for ensuring data and metadata are FAIR ([www.go-fair.org/fair-principles](http://www.go-fair.org/fair-principles)). Funders, journals, and policymakers are increasingly requiring the implementation of the FAIR principles for all research data and other related digital objects. Consideration of the FAIR principles should begin when a project application is written and continue throughout the data lifecycle.

## Implementing the FAIR principles

While the FAIR principles provide a framework, it remains the responsibility of researchers to decide how they will ensure their data and metadata are FAIR. Fortunately for researchers, there are materials that can be used for guidance to implement the FAIR principles at the project, group, and institutional levels (Table 1). However, the complexity of implementing the FAIR principles differs between analyses and data types. Researchers may lack time or funding, or awareness and understanding of the FAIR principles or the various initiatives that aim to support FAIR data practices.

**Implementing FAIR needs to be made easier:** To ensure widespread adoption of the FAIR principles, the threshold for implementation needs to be reduced substantially, such as with simple-to-use tools for easy data deposition and access via dedicated repositories. This is an area where national and international cardiovascular research societies, funding bodies, and publishing houses could drive progress for RDM in cardiovascular research. This may take the form of software solutions, such as the development of software that allows researchers to deposit specific sets of data (for example, the data for creating a figure) into a ‘data container’ for automated assignment to different data categories. These data could then be linked to the relevant published paper; readers could access the data by clicking on any figure in the paper (in HTML and PDF formats, for example). This would require significant expertise to develop, but comparable systems linking figures to data have been proposed [28].

**FAIR data are not necessarily ‘open’ data** According to the Open Data Handbook, open data are data that ‘can be freely used, reused, and distributed by anyone’ (<http://opendatahandbook.org/guide/en/what-is-open-data/>). The FAIR principles, in contrast, do not require data to be open. Although data should be findable and reusable, researchers may have good reasons to prevent public access of data, whether for privacy, national security, commercial, or intellectual property reasons (<https://www.go-fair.org/resources/faq/ask-question-difference-fair-data-open-data/>). However, researchers should nevertheless define a process by which data can be accessed and state the conditions under which data may be reused.

# Research Data Management Challenges in Cardiovascular Science

Whether working with approaches that build on biological reduction or biological integration, the huge volume and complexity of data that can be acquired with modern techniques limits data findability and requires dedicated infrastructure (data repositories, for example) if data are to be shared effectively. There are also currently few incentives for researchers to share their data.

## Managing data volume: sharing data via data repositories

**Which data should be shared for reuse?** While the idea behind *open* data may be to share all data [12], *FAIR* data sharing does not require sharing and storing everything forever. Data which are known to be flawed, due to experimental error for instance, may not need to be shared. The distinction between good-quality data and poor-quality data may not always be obvious, however, and will depend at least in part on subjective evaluation by researchers [18]. In addition, researchers should decide whether to share raw data or processed data. Research teams, institutions, and governments should define clear data-sharing policies to ensure that researchers understand which data should be shared and how.

**How should data be shared for reuse?** Data repositories are centralised storage spaces where datasets can be deposited for access and reuse by other users (although authorisation and authentication may be required) and are an important part of the data sharing infrastructure. There are several thousand data repositories, ranging from generalist repositories to specialist repositories for specific kinds of data.

**Choosing a data repository can be challenging:** Given the huge number of repositories, each with their own data standards and policies, researchers should take care when choosing a repository for their datasets. Services such as re3data (<https://www.re3data.org>) or FAIRsharing, a searchable interconnected registry of data standards, databases, and data policies across all research areas, provide a means to discover relevant repositories that meet FAIR requirements ([www.fairsharing.org](http://www.fairsharing.org/))[24]. International cross-discipline efforts have focused on the development of a minimal set of descriptive repository attributes, for example a common core set of metadata fields in a registry of repositories [29]. Providing these attributes in a human- and machine-accessible format, such as within FAIRsharing, can help researchers discover and select an appropriate repository. At minimum, repositories should automatically provide a globally unique and persistent identifier to every element of each dataset [30]. Identifiers should be persistent, even if data are moved from one repository to another, to prevent ‘link rot’ (a phenomenon in which hyperlinks tend to stop pointing to their original target page or file over time) and ensure continued access to the data for future research [30]. Repositories should also require deposition of sufficient metadata to allow other users to understand, process, and compare the data in a meaningful way. In the field of next-generation sequencing, the most common data portals (NCBI’s Sequence Read Archive and EMBL-EBI’s European Nucleotide Archive) require uploaded data to include detailed and well-defined metadata, including information on biological samples, experiments, and linked publications [31].

Some repositories are curated, providing additional services such as ensuring that submitted data function correctly, are adequately documented, and are deidentified or anonymised if relating to human beings. The Proteomics Identifications (PRIDE) Archive database, for example, is a centralised, curated, and standards-compliant public data repository for mass-spectrometry-based proteomics data (<https://www.ebi.ac.uk/pride/>) [32-35]. In all cases, however, researchers should be mindful that repositories can and do shut down, and investigators may need to re-upload data to another appropriate repository.

**Infrastructure is key:** Data use and reuse rely upon infrastructure at the local, national, and international levels. Given the increasing size of datasets, data reuse may also be severely limited by physical infrastructure, particularly data transfer speeds and available bandwidth. Data storage also has high energy and environmental costs, at least if it involves ‘spinning discs’ – which is considered the safest version of electronic data storage as continuous checking can be part of the process. To reduce storage costs, lossless data compression methods are needed. In the case of sequencing data, the CRAM data format files are up to 50% smaller compared to the BAM format (and even smaller compared to the original FASTQ format) without losing information present in the raw data [36].

While initiatives like the European Open Science Cloud (EOSC; <https://eosc-hub.eu/>) may one day provide infrastructure to support open science practices, more immediate improvements to physical infrastructure are largely out of individual researchers’ hands and require significant investment by institutions and charitable or governmental organisations. In Germany, the National Research Data Infrastructure (*Nationale Forschungsdaten Infrastruktur*, NFDI, <https://www.nfdi.de/>) aims to create a permanent digital repository of knowledge in which datasets from science and research are systematically accessed, networked, and made usable. The UK Biobank prospective cohort is a continuously expanding resource with large-scale and in-depth phenotyping and genomic data that is primarily funded by Medical Research Council and Wellcome [37].

Funding for development and maintenance of new or existing data sharing infrastructure is likely to be significant and support from government bodies and large charitable organisations is usually necessary. The UK Biobank, for example, has core funding of £180 million and is supported in part by the UK Department of Health, the National Institute for Care and Research, and Medical Research Council, as well as major charities including the Wellcome Trust, the British Heart Foundation, and Cancer Research UK. Similarly, the NFDI is supported by the German Federal Government and the individual German states, and the EOSC builds on infrastructure supported by the European Commission and EU member states.

**Improving secure access to data:** A federated and secure approach to data access, for example through an identification system that allows researchers to securely access data repositories from anywhere in the world (comparable to Eduroam for wireless internet access) may also reduce the barriers to legitimate access and reuse of data, while ensuring that only approved users are able to access data. Such a scheme would require the involvement of researchers, academic institutions, and the repositories themselves, as well as possibly involving national and international government bodies.

## Encouraging data sharing

**Provide incentives to share data:** Journals are increasingly requiring authors to include a link in their papers that directs readers to the repository hosting the relevant dataset. Publications that are linked to the underlying research data are cited more often in an effect akin to the open-access citation advantage [13,38]. However, if data repositories are to realise their potential, it will be necessary to further incentivise contribution. Measures of scientific productivity, and the associated academic recognition and rewards, remain focused on the number of publications and citation rates in academic journals, rather than the collection and sharing of data [39-40]. Researchers who share well-annotated datasets via repositories should be recognised and rewarded by funding bodies and universities in a suitable manner [41-42].

Although most researchers may be willing to share data with other researchers [8], they may lack the time, resources, and skills to do so effectively [18]. In these cases, institutions should ensure adequate funding, training, and education are available. Researchers who do share their data should set an example to their peers by including a link to repository data in any articles that use those data; this will also help increase the findability of related datasets and thus reduce one common barrier to access, while helping to further normalise data sharing [43]. Researchers should also work with their community to define standards and expectations for the management, sharing, and reuse of data and associated metadata. At least initially, this will take time – which is already scarce ­– away from research, but action can be a simple case of joining an already existing initiative such as one of the many working and interest groups within the Research Data Alliance (<https://rd-alliance.org/>).

**Cite data, not just publications:** Some researchers may be hesitant to share data for fear of other researchers using the data for their own publications without the original investigators being credited, or worse, of being ‘scooped’ or having their data misrepresented [44,8]. Publication embargos give researchers the chance to publish their findings before data are widely shared; however, embargos may put unnecessary time pressure on research groups to publish within short timeframes and may unintentionally delay bona fide analysis by other research groups [40]. Some researchers choose to include the original investigators as co-authors on any publication that results from data reuse [45,46]; however, this practice may not align with how journals decide who or who does not qualify as an author (particularly if the original investigator has only contributed data). It also raises the question of how many original investigators should be included, and it may create difficulties if the original investigators disagree with the new methods, findings, or conclusions [39,40,45,47]. An alternative may be to ensure that data are credited or cited wherever they are used or reused. In the interests of fairness and transparency, journals and publishers should require authors of publications that reuse data to credit the investigators who collected the original data and cite the original dataset [39,41,42]. Data-source crediting would allow some academic recognition and reward for data sharing, and help researchers satisfy funding obligations to share their data [40]. However, such a system would require a standardised format for crediting data that can be used and recognised by journals, academic institutions, and funding bodies. Some work has been done to define such a system: the Joint Declaration of Data Citation Principles recommends the citation of primary data in the same way that literature is cited [41,48], and Pierce and colleagues have proposed an alternative system in which all publications include a link to the underlying dataset via citation of a persistent data identifier that has been issued by a data repository and is linked to ORCID identifiers of the individuals who collected, analysed, and shared the original data [39,42]. Whatever form it takes, the adoption of any data-crediting system will require commitment from authors, journals, and funding bodies [40].

# Data Challenges Arising From Approaches Building on Biological Reduction

Cardiovascular research involving biological reduction aims to study complex systems like the heart by focusing on their constituent elements, including genes, proteins, cell types, and their functions. Researchers may choose to share and combine the resulting data within multi-disciplinary consortia, such as the EU-CardioRNA COST Action network (<http://www.cardiorna.eu/>)[49] or the Encyclopedia of DNA Elements (ENCODE; <https://www.encodeproject.org>)[50]. However, combining datasets is difficult, and for many data modalities (such as electrophysiology or mechanics measurements) there may be no established processes or structures to support it.

## Managing data heterogeneity: standardisation and harmonisation

Sharing and combining datasets can be challenging due to the heterogeneity of the data involved, particularly if data are obtained using team-specific protocols and with limited standardisation across laboratories. It is important that effective guidance and support for data handling is provided by research teams and institutions to prevent data handling from being conducted in a fragmented and inconsistent manner within individual projects or by individual researchers [18].

**Standardised terminology:** Comparability of data can be restricted by a lack of standardisation in vocabulary, terminology, and coding schemes; even simple parameters such as ‘age’ may be recorded to the nearest day, week, month, or year by different groups, using various units and annotations. Some global standards exist, including the International Organization for Standardization ISO standards, but these are not always adhered to. In addition, the diverse data types involved in cardiovascular research mean that more specific guidelines are needed when collecting and reporting data. Some standardised terminologies for life science experiments are already available, including the HUGO Gene Nomenclature Committee guidelines for human gene names and the Ontology for Biomedical Investigations (<https://obi-ontology.org/>)[51], which defines terms for various assays, devices, and materials, but greater standardisation of terminology (and better adherence to existing standards) is needed across cardiovascular science.

**Standardised protocols and analysis:** Beyond terminology, standardised collection, processing, and analysis pipelines are also needed to ensure interoperability and comparability of data. In mature fields such as transcriptomics, standards, guidelines, and resources that collect standards (see FAIRsharing’s transcriptomics resources) may be the norm. There may also be computational frameworks for data processing and handling that researchers can take advantage of, such as BioImageIT and the Galaxy Project [52,21]; the Galaxy Project, for example, is a scientific workflow system that allows storage of information such as the software and database versions used, as well as the applied parameters, thereby aiding the reproducibility of analyses. In other areas, there are multiple standards or guidelines that may need to be harmonised top-down, driven by key opinion leaders. In evolving fields, such as single-cell transcriptomics, however, there is a need to develop and adopt a community-wide standard for the collection and preservation of data and metadata ‘on-the-job’. This may be best accomplished bottom-up, with community-driven approaches. In either case, consensus is required, and standards need to evolve with technology. Agreeing on and maintaining shared standards is likely to be challenging and requires continued, committed effort.

**Standardised quality assessment:** Combining datasets ultimately depends on the quality, completeness, and validity of the data being shared. Sample quality is an important but often overlooked element of research, particularly when dealing with raw data from approaches building on biological reduction. Poor-quality samples can lead to poor-quality or even invalid data, which may be especially difficult to detect and exclude once data have been pre-processed, particularly if those reusing the data are unable to determine how data were collected or how sample quality was assessed. It is therefore important that poor-quality samples are excluded from analyses before they may be shared with others. This process is necessarily somewhat subjective, so standardised metrics and assessment protocols for sample quality may be needed [53].

Quality assessment of samples is also a prerequisite for translating research findings to clinical applications. Using RNA sequencing as an example, data quality and the reproducibility of findings are highly influenced by sample quality, since RNA molecules are relatively unstable as initial material for several downstream applications, and adequate standard operating procedures should be followed for sample (i.e., biological fluid or tissue) collection, handling, storage, RNA extraction, and quality control. Potential clinical applications for both circulating RNA-based biomarkers and quantitative PCR have been hindered by a lack of technical standardisation. Similarly, poor quality control of purified protein samples can lead to irreproducible or misleading downstream observations and attempts to standardise quality assessment of protein samples have been advanced [54]. Groups such as the EU-CardioRNA consortium are helping to define harmonised, standardised guidelines for RNA handling [55-58]. Professional biobanks, set up as core facilities in many university medical centres around the world, also offer support regarding standardisation of sample processes and quality control [59].

The importance of quality assessment as part of responsible RDM can also be seen in the context of imaging data. Quantitative imaging (electron and light microscopy) is now one of the main experimental and pre-clinical research modalities in cardiovascular science. The drive to develop approaches with ever higher throughput means more imaging data than ever are produced and made available, and this requires a unique set of sample quality assurances. Parameters such as time of sample preservation in relation to organ excision or cell isolation should be recorded. In the case of time of sample preservation, cardiac tissue and cells remodel rapidly ex vivo, which could lead to erroneous conclusions regarding sub-cellular structure. For example, over 50% of cardiomyocyte caveolae disappear within 8 hours after cell isolation, affecting cellular nano-domain architecture and changing membrane capacitance [60].

**Reporting guidelines:** The adoption of community reporting guidelines (or minimum information standards) that describe how to report everything from sample quality to the data processing protocols used can facilitate data sharing, streamline workflows, and allow for the long-term preservation of and access to information [26,43,61].

Guidelines and tools for reporting experimental data already exist in several areas of life science and clinical research, including metabolomics (<https://github.com/MSI-Metabolomics-Standards-Initiative/CIMR/>) proteomics (<https://www.psidev.info/miape>), high-throughput nucleotide sequencing [62], single-cell RNA sequencing [19,63,64], description of biospecimen quality [65], and animal research [66]. BioCompute ([https://www.biocomputeobject.org](https://www.biocomputeobject.org/)) is an example of a community-driven framework for the standardised reporting of data [67], whereas the Global Alliance for Genomics and Health (GA4GH; [www.ga4gh.org](http://www.ga4gh.org)) is developing standards for responsibly sharing genomic data, and has recently defined the ‘Phenopacket’ schema as a way to share clinical longitudinal phenotypic information about patients [68]. Initiatives like the OMOP Common Data Model (<https://www.ohdsi.org/data-standardization/>) can be used to standardise the format and content of observational data across databases, allowing for evidence generation using data from a wide variety of sources. However, many researchers may not adopt or adhere to reporting guidelines due to limited time, funding, or awareness [8,18].

To avoid wasting time and resources re-creating existing initiatives and standards, it is important that researchers first determine whether an existing set of tools or standards suits their needs before starting their own initiatives. One way to accomplish this may be for funding bodies to require an evaluation of suitable tools or standards for successful applications. Existing standards can be explored within the FAIRsharing standards registry (<https://fairsharing.org/standards>), while registries such as bio.tools (<https://bio.tools/>) provide information about software tools, databases and services for bioinformatics and the life sciences. The use of complex standards can be time-consuming to learn, however, and since most research sites lack a documentary workforce, appropriate tools for simplified working with reporting guidelines are needed.

Where reporting standards do not exist, Crystal-Ornelas and colleagues have formulated guidelines for research communities who wish to self-organise and create community-centric reporting formats for data and metadata, from the identification of existing standards and best practices to the creation of documentation and training resources [26].

**Better standardisation and sharing of data are possible:** The lack of standardisation in data collection, processing, and analysis means that data are increasingly heterogeneous, limiting comparability and reusability. However, the challenges are not insurmountable: large-scale collaborative initiatives like the National Sleep Research Resource ([www.sleepdata.org](http://www.sleepdata.org)) and the UK Biobank (<https://www.ukbiobank.ac.uk>) have shown it is possible for researchers to organise and collaborate on the collection and sharing of large volumes of health data for their mutual benefit, despite the challenges. Although it is important to recognise that there is unlikely to be a ‘one-size-fits-all’ solution, these initiatives may provide a model for similar efforts in cardiovascular science.

# Data Challenges Arising From Approaches Building on Biological Integration

Approaches to cardiovascular research involving biological reduction have led to significant advances in our understanding of specific systems within the heart. However, putting these findings into a wider biological context requires ‘integrative’ approaches. These involve combining information from multiple disciplines, for example linking genome sequencing data with basic and clinical functional and structural data, or computational modelling data.

## Identifying meaningful, actionable data: functional studies

The increasing availability and adoption of longitudinal monitoring and mHealth technologies means that large volumes of functional data can now be collected at a patient level via smartphones and other personal electronic devices [69,70]. However, much of this information is underused in integrative studies for various reasons (e.g., the data are of insufficient quality for scientific analyses or are not comparable with data obtained using older techniques). Given the volume of data available, it may also be difficult for researchers to identify meaningful and actionable data for clinical applications.

**Data may not always accurately reflect disease status:** Functional data, such as vital signs, are routinely collected longitudinally in clinics and may be used to categorise patients by disease status or determining the severity of disease. However, the data may not accurately represent complex pathophysiological mechanisms that underlie disease and may not efficiently utilise the full spectrum of data sources that are available [71]. In the case of sleep-disordered breathing, for example, functional data are often collected for a single night only, ignoring night-to-night variability. Therefore, the apnoea-hypopnoea index, reflecting the number of apnoeas and hypopneas per hour sleep, may not accurately represent the severity of sleep apnoea [71]. However, new technologies, including mHealth, may allow researchers to adopt a more complete, disease- and patient-oriented approach using more dimensions of time-series data, for example. Patients are increasingly collecting their own data via mHealth devices, including smartphones and smartwatches [70]. These devices represent valuable data sources for integrative approaches, but the quality of the data they provide in a clinical setting is reliant on patient adherence and cooperation [72,73]. Patient education on the use of mHealth technology may help improve motivation and adherence, and thus improve the quality and quantity of data collected.

**Identify and focus on meaningful, actionable data:** Given the volume of data that can be collected with technologies like mHealth or modern high-throughput techniques, it is important that researchers focus on data that are meaningful (e.g., data that are relevant to the disease being studied) and actionable (e.g., data that can be used to inform a specific treatment decision). In a clinical setting, structured care pathways (healthcare management plans for specific groups of patients) can be used to select appropriate mHealth technologies, educate patients on their use, and agree on a mHealth monitoring plan [74]. Collaborations between scientific researchers, healthcare providers, manufacturers, software developers, and insurance companies may provide an opportunity to influence and guide the development of new devices, common interfaces, and software to improve the quality and utility of data collected and how those data are integrated with other clinical and scientific data. However, data security, including deidentification or anonymisation where necessary, should remain a priority when working with mHealth data [75]. New technology (whether mHealth or otherwise) is also often introduced without information on internal data processing algorithms or sufficient guidance on how the resulting data should be interpreted or integrated into treatment plans. Better guidance from manufacturers is needed to ensure researchers and clinicians can effectively use the most appropriate available technologies, better identify useful meaningful and actionable data, and improve treatment decision-making and risk assessment.

## Computational modelling: the importance of raw experimental data

Computational modelling allows cardiovascular researchers to combine large amounts of data into mechanistic or phenomenological biophysical models. For example, simulations of cardiac electrophysiology are being used to improve our understanding of the mechanisms of atrial fibrillation and the action of pharmacological interventions and thus improve clinical care [76].

**Raw data are essential for calibration and validation:** Computational models rely on raw data for model conceptualisation, generation, calibration, and validation. However, as raw data of the required quality are often unavailable, calibration of models typically relies on published ‘summary’ data (or, often, other pre-existing models, based on data from various species [77,78]. A lack of model validation can result in overfitting and poor predictive capabilities and is not acceptable for real-world application of these models; greater availability and accessibility of raw experimental data may allow for easier and improved model calibration and validation.

**Model benchmarking:** For a given system, there may be many models available – some more and some less well-suited to any given application – and it can be difficult for researchers to choose or compare them. Better benchmarking (structured comparisons based on pre-defined metrics) and better curation of experimental data may facilitate the convergence of model structures. This has been attempted, led by models from the cardiovascular field, using mark-up language-based model repositories, such as CellML (<https://www.cellml.org/>). These repositories not only accumulate published models and ‘translate’ them into an Esperanto-like format, but also offer curation and backwards-compatibility testing using automated benchmarking that runs in the background of the model repository. This approach has been highly influential and is being extended from cell to tissue and other levels of systems integration [79].

# Other challenges

## Data security, patient consent, and increasing patient control of data

**Legal aspects of sharing sensitive data:** Sharing sensitive data is rightfully strictly regulated, but levels of regulation differ internationally, which can make the sharing of data with researchers outside the EU difficult. This difficulty is justified given the risks associated with improper sharing of personal data, but nevertheless it presents a challenge when researchers wish to work together within international consortia. The EU’s General Data Protection Regulation (GDPR), for example, applies to the processing of personal information, including data that may be used in biomedical research. Health data are considered ‘sensitive data’ and requires the highest level of protection [74,80,81]. While GDPR represents a significant harmonisation of EU data protection laws, it has generated discussion and confusion among researchers as to how and when it applies. Education is needed to ensure that researchers understand when and how data may be shared, what researchers need to do to ensure that they are in compliance with GDPR and other applicable laws, and what technology is available to allow data sharing while maintaining security (e.g., via federated machine learning). But even if data sharing regulations are strictly adhered to, advances in image processing algorithms may allow identification of individuals from formally anonymised data, such as MRI images [82], which highlights the need for caution and the limits of personal data protections approaches as they currently stand.

**Improving data security through decentralised machine-learning techniques:** Machine learning involves the use of algorithms to build predictive models based on large sets of sample or ‘training’ data and provides an opportunity to process and analyse data at an unprecedented rate and at great depth and may allow for better detection and risk prediction of cardiovascular problems such as heart failure [83]. In ‘local’ learning methods, training models use data from a single centre; this is the most secure approach as data are not shared with other centres. Central learning involves sharing raw data between centres via a dedicated server; this allows models to access a larger set of training data, but also means data are at greater risk of being intercepted. In clinical studies, the training data being shared may include sensitive patient health information. Patient confidentiality concerns may be partially addressed through decentralised machine learning techniques, such as federated or swarm learning via systems such as Personal Health Train and DataShield [84,85]. Federated learning involves local training of algorithms, with only parameters (e.g., from a neural network) being shared to a dedicated server; this is more secure than central learning as no raw data are shared between centres. Swarm learning uses technologies such as blockchain to further decentralise machine learning and may represent an even more secure alternative to federated learning [86].

**Giving patients control of their data:** Providing patients with control of their data may also improve data security. The Personal Medical Safe (PROMISE) research project, for example, gave patients control of their own genomic data via a mobile app, allowing them to accept or decline requests to use their genomic data in studies via the app [87]. Most patients felt the app was a suitable solution to the sensitivity of genomic data and reported satisfaction at being in control of their own data. This approach is not without risk however, particularly if patients do not feel confident making decisions about how their data should be used; several patients in the study reported feeling afraid of being responsible for their own data [87]. This approach would also require greater efforts to inform and educate patients about the research questions for which their data may be requested.

**Managing patient consent data:** In research involving patients, consent must be managed and documented appropriately. Patient intent is often to allow sharing of their data if it will improve diagnostic and therapeutic options [88,89], but lack of information on the exact parameters of consent may prevent reuse of data where consent exists but is not easily traceable. Digitisation and automation of (remote) patient consent is increasing and may help to improve access to samples and data as details of patient consent can be more efficiently traced. Improved training of physicians with regards to consent and sensitive data is needed. The most desirable option would be a general ‘broad consent’ that is part of patient admission protocols; the general adoption of such a standard in German hospitals is unlikely in the short- to medium-term [90], although templates have been developed for this purpose by several initiatives (<https://www.akek.de/biobanken/>; <https://www.medizininformatik-initiative.de/de/mustertext-zur-patienteneinwilligung>).

Managing and documenting patient consent for organ donation is particularly challenging as practice and legal restrictions can vary significantly by jurisdiction. Countries may operate opt-in (e.g., Germany) or opt-out systems (e.g., Spain and Austria), and may or may not require explicit consent by the donor and/or their family. There may also be limits on how donated organs may be used. In the United Kingdom, for example, donated organs and tissues that are not suitable for transplantation may be used for research with the permission of the donor or their family (<https://www.organdonation.nhs.uk/helping-you-to-decide/about-organ-donation/consent/>). In Germany, however, organ donation is exclusively intended for medical use in transplantation, and excludes otherwise typical applications such as training, quality control, and research. The fact that this tissue must be discarded, despite being uniquely valuable for research, also poses a significant RDM problem as it contributes to a lack of control data in human beings – the very species for whom biomedical research is conducted. This raises a serious question about the scientific and ethical costs of *not* using human healthy cardiac tissue in research, particularly when a donor agreement is available and a transplant is not possible for other reasons (medical, logistic, etc.), something that occurs for between 20% and 70% of organs donated in Germany [91].

## The societal costs of cardiovascular research

**Data sharing and FAIR research help offset the societal cost of scientific research:** Scientific research comes at a cost to society: taxpayers, industry, and private donors provide funds for research projects; patients provide samples and sensitive personal data; research animals must be bred, cared for, and sacrificed; time and resources are spent on the education and training of scientists and clinicians; infrastructures must be set up to allow experimental and clinical research. The financial costs of non-FAIR data can be quantified: a study by the European Commission estimated that not having FAIR research data costs €10.2 billion per year in Europe, with an additional estimated €16 billion impact on innovation; it is worth mentioning that these figures do not include the non-quantifiable benefits of making data FAIR [92]. The societal cost of research, including the burden on taxpayers, can be partly offset by more efficient use of data, particularly through data sharing and reuse. Making data FAIR will increase the value of the data obtained, potentially accelerate progress in the improvement of therapeutics and diagnostics, and maximise the return on investment for funders. However, data reuse is not without costs, including the time required for RDM, data sharing, dataset evaluation, and dataset merging.

# Who Is Responsible for RDM in Cardiovascular Science?

Effective RDM in cardiovascular science requires committed action at all levels, from researchers to health authorities.

**Researchers:** Researchers are ultimately responsible for ensuring that research data are suitably managed and shared according to the FAIR principles. This includes ensuring that data are adequately documented and made available for reuse as appropriate. Researchers may also be responsible for effective RDM in a more direct way: many funding bodies require researchers to have a data management plan as a condition of funding [8]. Regardless of whether individual funders require such plans, funding bodies are likely to have higher expectations with regards to the scientific value and impact of the research they support given the highly competitive global research environment at present; it is therefore in the interests of researchers to take the necessary steps to ensure that their data has the greatest impact and utility. Researchers should also aim to set a good example to their peers (and the next generation of scientists, though hopefully they will be the emerging drivers of modern-day RDM) by making data available where possible, and citing or crediting data sources.

**Data stewards:** Although researchers are ultimately responsible for the management of their data, some institutions employ dedicated data stewards [9]. Data stewards work in collaboration with the data acquirers and users to ensure that data, including metadata, are fit for purpose. Data stewards can provide advice to researchers on every step of the data lifecycle and help ensure that data are handled in an appropriate manner that is compliant with local and national policies, community standards and guidelines, and principles such as FAIR.

However, data stewards are not currently part of standard cardiovascular research infrastructures. Even where present, they cannot alone be responsible for ensuring data are obtained, analysed, and shared appropriately. Data stewards also cannot be solely responsible for training data generators and data users; this task should be performed by the institutions responsible for general training and education, with data stewards providing domain-specific education. In addition, researchers may not be aware of data stewards or their capabilities; institutions should ideally provide funding, education, and training so that data stewards can be integrated into research.

**Journals and publishers:** Journals and publishers should require authors to include links to all relevant raw or processed data, metadata, and other relevant materials in their submissions when publicly available (for example using the RO-Crate format). Where data are not publicly available, data sharing statements should indicate how the data may be accessed or requested, and authors or organisations should be expected to make data available upon request. Links to appropriate software repositories and archives of particular versions of software should also be considered for inclusion according to the recommendations of the community, such as the RDA FAIR4RS WG [93], the Software Sustainability Institute ([https://www.software.ac.uk/https://www.software.ac.uk/](https://www.software.ac.uk/)), and Software Heritage ([https://www.softwareheritage.org/https://www.softwareheritage.org/](https://www.softwareheritage.org/)). Journals should also take greater responsibility for confirming that submitted materials include working links to the raw data, metadata, and other relevant materials, ideally via a persistent identifier (like the digital object identifier or DOI). However, given the limited resources of many journals (and the high burden already placed on the peer-review process), the ultimate responsibility will continue to lie with authors to provide working links at submission. This calls for lower-threshold tools for data deposition and access, which will need to be funded, developed, and adopted. Publishers should also recognise the importance of negative and inconclusive data and should ensure that they are seriously considered for publication in peer-reviewed journals.

**Research institutions, universities, funding bodies, and government organisations:** Universities, funders, and government bodies must recognise and reward the collection and sharing of data in the same way that they recognise and reward publication activity. They should also support effective RDM, including data sharing and metadata documentation, via training and education and by defining and implementing data sharing policies. Universities, funders, and government bodies should also be aware of the need to provide sufficient long-term funds for data storage and sharing; this includes funding, improving, and future-proofing repositories, as well as the infrastructure and personnel needed for data storage, sharing, and reuse (e.g., funding data stewards). Although this may seem expensive, the scientific, societal, educational, ethical, and financial costs of not sharing data are likely to be much higher. There is also a need to establish data management training modules at graduate level in research institutions, with more advanced training provided at post-doctoral level. There is a chance to accelerate progress in Germany regarding the education of physicians in terms of the collection, use, quality assessment, and analysis of data via updates to the *Approbationsordnung.*

Coordination of RDM practices remains a challenge. Currently, the German Research Foundation (*Deutsche Forschungsgemeinschaft*, DFG) encourages RDM policy development by each ‘network grant’. Whether this is the most effective way forward remains to be seen, as parallel work, at times even within one university or faculty, would seem counterproductive. However, the diversity of RDM solutions implemented in different consortia (e.g., Collaborative Research Centres), institutions (e.g., the European Molecular Biology Laboratory) and in established public databases (such as the NCBI and European Bioinformatics Institute) mean that many different solutions for RDM are tested. It will be important to consolidate these efforts, which will require a considerable investment of time and expertise. Standardisation of RDM policy across consortia, institutions, and fields of research would be a welcome step forward.

Finally, while the legal hurdles that need to be overcome when sharing data between, for example, EU- and non-EU-based research teams may be in part mitigated with technical solutions, a definitive solution will require the involvement of national funding bodies and governmental entities.

# Conclusions

Data reuse should be factored in at every stage of scientific research, and researchers should foster a culture of open, FAIR science, through sharing good-quality, well-annotated data and metadata in repositories, defining and following agreed-upon standards, crediting and linking to the data of others, and publishing negative results. Researchers should make use of resources such as FAIRsharing (<https://fairsharing.org/>) and lobby their institution, funders, or colleagues to set out and adhere to policies, reporting guidelines, and tools that are relevant to their discipline.

The cardiovascular research community should also work to make sharing raw data and metadata the norm at all levels; this may require the definition of minimum information standards for use in cardiovascular research [94]. Moreover, it requires incentives to adopt cardiovascular reporting guidelines once they exist (an example of a well-intentioned and broadly endorsed – yet under-utilised – reporting guideline is MICEE [95]). As much cardiovascular research data and accompanying metadata should be made available via public repositories as possible, with the ultimate goal being the long-term and sustainable storage and reuse of data.

Community-driven standardisation and harmonisation at all stages of the data lifecycle is needed to reduce the heterogeneity of data and ensure good data quality. This standardisation may be top-down or bottom-up, depending on the maturity of a given field of research. Standards should be considered living documents that need to be regularly adapted to new technologies or methods. However, it is important to recognise that there is unlikely to be a one-size-fits-all solution for effective RDM in cardiovascular science. The development, adoption, and application of RDM practices will require careful consideration at all levels and in all areas of cardiovascular research and should be part of the new *Ärztliche Approbationsordnung*.

To ensure the next generation of researchers adopt best practices, training and education needs to start early (ideally at the undergraduate level); for example, the teaching of Good Clinical Practice should introduce the principles and responsibilities of data management. These measures will require dedicated and committed efforts at all levels. Ethical questions, including the issue of the underutilisation of donated organs, should be raised with the *Ethikrat* (Ethics Council) in Germany by the DGK and the DZHK.

Institutions, as well as state and national government bodies and publishers, should commit to improving and future-proofing digital infrastructures for data storage and sharing, including funding librarians, data stewards, and IT support personnel. Institutions should also encourage education and training programmes around data generation, data use, data quality, data analysis, and evaluation and reuse of existing datasets. This requires concerted investment into the development of low-threshold tools for data and metadata sharing during data acquisition (electronic records), processing (automated metadata annotation), and publication (low-level access to key data, such as contained in figures, via a ‘data container’).

It is not enough to simply recognise the importance of responsible and effective RDM: it must be put into practice. To enable this, the authors encourage their professional societies and research organisations (including the DGK and DZHK), as well as funding and regulatory bodies, to spearhead a number of initiatives, including:

(i) supporting initiatives and/or lobbying national funding bodies to aid a more concerted effort to develop relevant RDM processes and tools and FAIR data sharing approaches across the life sciences, including the development of and adherence to minimum reporting guidelines;

(ii) developing a generic (PDF- or HTML-compatible), pragmatic (focussing on data used to create figures in peer-reviewed publications), low-threshold (data container) tool to make a first but definitive step to data sharing that is independent of the research subject, methods used, and level of investigation involved; and

(iii) pushing for generalised ‘point-of-entry’ consenting of patients for the use of their data and any biological materials acquired in the process of diagnostic or therapeutic interventions that would otherwise be discarded, and probing the ethical acceptability of discarding healthy human donor tissue (*the* biological reference) that may not be used clinically (which must remain the primary aim of all donor organ utilisation).

References

1. Kohl P, Noble D (2009) Systems biology and the virtual physiological human. Mol Syst Biol. 5:292. doi: 10.1038/msb.2009.51
2. Holdren JP (2013). Increasing access to the results of federally funded scientific research. Office of Science and Technology Policy. Executive Office of the President. USA. Available at <https://obamawhitehouse.archives.gov/blog/2016/02/22/increasing-access-resultsfederally-funded-science>. Accessed Nov 22, 2022
3. Organization for Economic Co-Operation and Development (2004). Declaration on access to research data from public funding. Available at <https://legalinstruments.oecd.org/en/instruments/157>. Accessed Nov 22, 2022.
4. Taichman DB, Sahni P, Pinborg A, Peiperl L, Laine C, James A, Hong ST, Haileamlak A, Gollogly L, Godlee F, Frizelle FA, Florenzano F, Drazen JM, Bauchner H, Baethge C, Backus J (2017) Data Sharing Statements for Clinical Trials - A Requirement of the International Committee of Medical Journal Editors. N Engl J Med. 376(23):2277-2279. doi: 10.1056/NEJMe1705439
5. Federer LM, Belter CW, Joubert DJ, Livinski A, Lu YL, Snyders LN, Thompson H (2018) Data sharing in PLOS ONE: An analysis of Data Availability Statements. PLoS One. 13(5):e0194768. doi: 10.1371/journal.pone.0194768
6. Stall S, Yarmey L, Cutcher-Gershenfeld J, Hanson B, Lehnert K, Nosek B, Parsons M, Robinson E, Wyborn L (2019) Make scientific data FAIR. Nature. 570(7759):27-29. doi: 10.1038/d41586-019-01720-7
7. Gewin, V (2016) Data sharing: An open mind on open data. Nature 529:117–119. doi : 10.1038/nj7584-117a
8. Tenopir C, Rice NM, Allard S, Baird L, Borycz J, Christian L, Grant B, Olendorf R, Sandusky RJ (2020) Data sharing, management, use, and reuse: Practices and perceptions of scientists worldwide. PLoS One. 15(3):e0229003. doi: 10.1371/journal.pone.0229003
9. Jansen P, van den Berg L, van Overveld P, Boiten JW (2018) Research Data Stewardship for Healthcare Professionals. In: Kubben P, Dumontier M, Dekker A (eds) Fundamentals of Clinical Data Science. Springer, Cham, Switzerland, pp.37–53
10. Nature (2018) Everyone needs a data-management plan. Editorial. Nature. 555(7696):286. doi: 10.1038/d41586-018-03065-z
11. Schiermeier Q (2018) Data management made simple. Nature 555:403-405. doi: 10.1038/d41586-018-03071-1
12. Dijkers MP (2019) A beginner's guide to data stewardship and data sharing. Spinal Cord. 57(3):169-182. doi: 10.1038/s41393-018-0232-6
13. Colavizza G, Hrynaszkiewicz I, Staden I, Whitaker K, McGillivray B. The citation advantage of linking publications to research data. PLoS One. 2020 Apr 22;15(4):e0230416. doi: 10.1371/journal.pone.0230416
14. Begley CG, Ellis LM (2012) Drug development: Raise standards for preclinical cancer research. Nature 483: 531–533
15. Mobley A, Linder SK, Braeuer R, Ellis LM, Zwellig L (2013) A survey on data reproducibility in cancer research provides insights into our limited ability to translate findings from the laboratory to the clinic. PLoS ONE. 8(5):e63221. doi: 10.1371/journal.pone.0063221
16. Prinz F, Schlange T, Asadullah K (2011) Believe it or not: how much can we rely on published data on potential drug targets? Nat Rev Drug Discov 10: 712. doi: 10.1038/nrd3439-c1
17. Ulrich H, Kock-Schoppenhauer AK, Deppenwiese N, Gött R, Kern J, Lablans M, Majeed RW, Stöhr MR, Stausberg J, Varghese J, Dugas M, Ingenerf J (2022) Understanding the Nature of Metadata: Systematic Review. J Med Internet Res. 24(1):e25440. doi: 10.2196/25440
18. Perrier L, Blondal E, MacDonald H (2020) The views, perspectives, and experiences of academic researchers with data sharing and reuse: A meta-synthesis. PLoS One. 15(2):e0229182. doi: 10.1371/journal.pone.0229182. Erratum in: PLoS One. 2020 Jun 2;15(6):e0234275
19. Puntambekar S, Hesselberth JR, Riemondy KA, Fu R (2021) Cell-level metadata are indispensable for documenting single-cell sequencing datasets. PLoS Biol. 19(5):e3001077. doi: 10.1371/journal.pbio.3001077
20. Nekrutenko A, Taylor J (2012) Next-generation sequencing data interpretation: enhancing reproducibility and accessibility. Nat Rev Genet 13(9):667-72. doi: 10.1038/nrg3305
21. Galaxy Community (2022). The Galaxy platform for accessible, reproducible and collaborative biomedical analyses: 2022 update. Nucleic acids research, 50(W1), W345–W351. Advance online publication. doi: 10.1093/nar/gkac247
22. Soiland-Reyes S, Sefton P, Crosas M, Castro LJ, Coppens F, Fernández JM, Garijo D, Grüning B, La Rosa M, Leo S, Ó Carragáin E, Portier M, Trisovic A, RO-Crate Community, Groth P, Goble C (2022) Packaging research artefacts with RO-Crate. Data Science 5(2) doi: 10.3233/DS-210053
23. Sarkans U, Chiu W, Collinson L, Darrow MC, Ellenberg J, Grunwald D, Hériché JK, Iudin A, Martins GG, Meehan T, Narayan K, Patwardhan A, Russell MRG, Saibil HR, Strambio-De-Castillia C, Swedlow JR, Tischer C, Uhlmann V, Verkade P, Barlow M, Bayraktar O, Birney E, Catavitello C, Cawthorne C, Wagner-Conrad S, Duke E, Paul-Gilloteaux P, Gustin E, Harkiolaki M, Kankaanpää P, Lemberger T, McEntyre J, Moore J, Nicholls AW, Onami S, Parkinson H, Parsons M, Romanchikova M, Sofroniew N, Swoger J, Utz N, Voortman LM, Wong F, Zhang P, Kleywegt GJ, Brazma A (2021) REMBI: Recommended Metadata for Biological Images—enabling reuse of microscopy data in biology. Nat Methods 18(12):1418–1422. doi: 10.1038/s41592-021-01166-8
24. Sansone SA, McQuilton P, Rocca-Serra P, Gonzalez-Beltran A, Izzo M, Lister AL, Thurston M; FAIRsharing Community (2019) FAIRsharing as a community approach to standards, repositories and policies. Nat Biotechnol. 37(4):358-367. doi: 10.1038/s41587-019-0080-8
25. Gonzalez-Beltran AN, Masuzzo P, Ampe C, Bakker GJ, Besson S, Eibl RH, Friedl P, Gunzer M, Kittisopikul M, Dévédec SEL, Leo S, Moore J, Paran Y, Prilusky J, Rocca-Serra P, Roudot P, Schuster M, Sergeant G, Strömblad S, Swedlow JR, van Erp M, Van Troys M, Zaritsky A, Sansone SA, Martens L (2020) Community standards for open cell migration data. Gigascience. 9(5):giaa041. doi: 10.1093/gigascience/giaa041
26. Crystal-Ornelas R, Varadharajan C, O'Ryan D, Beilsmith K, Bond-Lamberty B, Boye K, Burrus M, Cholia S, Christianson DS, Crow M, Damerow J, Ely KS, Goldman AE, Heinz SL, Hendrix VC, Kakalia Z, Mathes K, O'Brien F, Pennington SC, Robles E, Rogers A, Simmonds M, Velliquette T, Weisenhorn P, Welch JN, Whitenack K, Agarwal DA (2022) Enabling FAIR data in Earth and environmental science with community-centric (meta)data reporting formats. Sci Data. 9(1):700. doi: 10.1038/s41597-022-01606-w
27. Wilkinson MD, Dumontier M, Aalbersberg IJ, Appleton G, Axton M, Baak A, Blomberg N, Boiten JW, Bourne PE, Bouwman J, Brookes AJ, Clark T, Crosas M, Dillo I, Dumon O, Edmunds S, Evelo CT, Finkers R, Gonzalez-Beltran A, Gray AJ, Groth P, Goble C, Grethe JS, Heringa J, 't Hoen PA, Hooft R, Kuhn T, Kok R, Kok J, Lusher SJ, Martone ME, Mons A, Packer AL, Persson B, Rocca-Serra P, Roos M, van Schaik R, Sansone SA, Schultes E, Sengstag T, Slater T, Strawn G, Swertz MA, Thompson M, van der Lei J, van Mulligen E, Velterop J, Waagmeester A, Wittenburg P, Wolstencroft K, Zhao J, Mons B (2016) The FAIR Guiding Principles for scientific data management and stewardship. Scientific Data. 3:160018. doi: 10.1038/sdata.2016.18
28. Liechti R, George N, Götz L, El-Gebali S, Chasapi A, Crespo I, Xenarios I, Lemberger T (2017) SourceData: a semantic platform for curating and searching figures. Nat Methods. 14(11):1021-1022. doi: 10.1038/nmeth.4471
29. Cannon M, Graf C, McNeice K, Chan WM, Callaghan S, Carnevale I, Cranston I, Edmunds SC, Everitt N, Ganley E, Hrynaszkiewicz I, Khodiyar VK, Leary A, Lemberger T, MacCallum CJ, Murray H, Sharples K, Soares E Silva M, Wright G, (Moderator) McQuilton P, (Moderator) Sansone S-A (2021) Repository Features to Help Researchers: An invitation to a dialogue. Zenodo. doi: 10.5281/zenodo.4683794. Available at: <https://zenodo.org/record/4683794>
30. McMurry JA, Juty N, Blomberg N, Burdett T, Conlin T, Conte N, Courtot M, Deck J, Dumontier M, Fellows DK, Gonzalez-Beltran A, Gormanns P, Grethe J, Hastings J, Hériché JK, Hermjakob H, Ison JC, Jimenez RC, Jupp S, Kunze J, Laibe C, Le Novère N, Malone J, Martin MJ, McEntyre JR, Morris C, Muilu J, Müller W, Rocca-Serra P, Sansone SA, Sariyar M, Snoep JL, Soiland-Reyes S, Stanford NJ, Swainston N, Washington N, Williams AR, Wimalaratne SM, Winfree LM, Wolstencroft K, Goble C, Mungall CJ, Haendel MA, Parkinson H (2017) Identifiers for the 21st century: How to design, provision, and reuse persistent identifiers to maximize utility and impact of life science data. PLoS Biol. 15(6):e2001414. doi: 10.1371/journal.pbio.2001414
31. Stoesser G, Sterk P, Tuli MA, Stoehr PJ, Cameron GN (1997) The EMBL Nucleotide Sequence Database. Nucleic Acids Res. 25(1):7-14. doi: 10.1093/nar/25.1.7
32. Deutsch EW, Csordas A, Sun Z, Jarnuczak A, Perez-Riverol Y, Ternent T, Campbell D, Bernal-Llinares M, Okuda S, Kawano S, Moritz RL,1 Carver JJ., Wang M, Ishihama Y, Bandeira N, Hermjakob H and Vizcaíno JA (2017) The ProteomeXchange consortium in 2017: supporting the cultural change in proteomics public data deposition. Nucleic Acids Res. 45(Database issue): D1100–D1106
33. Martens L, Hermjakob H, Jones P, Adamski M, Taylor C, States D, Gevaert K, Vandekerckhove J, Apweiler R (2005) PRIDE: the proteomics identifications database. Proteomics. 5(13):3537-45. doi: 10.1002/pmic.200401303. Erratum in: Proteomics. 2005 Oct;5(15):4046
34. Perez-Riverol Y, Bai J, Bandla C, García-Seisdedos D, Hewapathirana S, Kamatchinathan S, Kundu DJ, Prakash A, Frericks-Zipper A, Eisenacher M, Walzer M, Wang S, Brazma A, Vizcaíno JA (2022) The PRIDE database resources in 2022: a hub for mass spectrometry-based proteomics evidences. Nucleic Acids Res. 50(D1):D543-D552. doi: 10.1093/nar/gkab1038
35. Vaudel M, Burkhart JM, Zahedi RP, Oveland E, Berven FS, Sickmann A, Martens L, Barsnes H. PeptideShaker enables reanalysis of MS-derived proteomics data sets. Nat Biotechnol. 2015 Jan;33(1):22-4. doi: 10.1038/nbt.3109
36. Bonfield JK (2021) CRAM 3.1: advances in the CRAM file format. bioRxiv 2021.09.15.460485; doi: 10.1101/2021.09.15.460485
37. Caleyachetty R, Littlejohns T, Lacey B, Bešević J, Conroy M, Collins R, Allen N (2021) United Kingdom Biobank (UK Biobank): JACC Focus Seminar 6/8. J Am Coll Cardiol;78(1):56-65. doi: 10.1016/j.jacc.2021.03.342.
38. Ottaviani J. The Post-Embargo Open Access Citation Advantage: It Exists (Probably), Its Modest (Usually), and the Rich Get Richer (of Course) (2016) PLoS One;11(8):e0159614. doi: 10.1371/journal.pone.0159614. Erratum in: PLoS One. 2016 Oct 21;11(10 ):e0165166.
39. Bierer BE, Crosas M, Pierce HH (2017) Data Authorship as an Incentive to Data Sharing. N Engl J Med. 377(4):402. doi: 10.1056/NEJMc1707245
40. Devriendt T, Shabani M, Borry P (2021) Data Sharing in Biomedical Sciences: A Systematic Review of Incentives. Biopreserv Biobank. 19(3):219-227. doi: 10.1089/bio.2020.0037
41. Cousijn H, Kenall A, Ganley E, Harrison M, Kernohan D, Lemberger T, Murphy F, Polischuk P, Taylor S, Martone M, Clark T (2018) A data citation roadmap for scientific publishers. Sci Data. 5:180259. doi: 10.1038/sdata.2018.259
42. Pierce HH, Dev A, Statham E, Bierer BE (2019) Credit data generators for data reuse. Nature. 570:30-32. doi: 10.1038/d41586-019-01715-4
43. Goodman A, Pepe A, Blocker AW, Borgman CL, Cranmer K, Crosas M, Di Stefano R, Gil Y, Groth P, Hedstrom M, Hogg DW, Kashyap V, Mahabal A, Siemiginowska A, Slavkovic A (2014) Ten simple rules for the care and feeding of scientific data. PLoS Comput Biol. 10(4):e1003542. doi: 10.1371/journal.pcbi.1003542
44. Longo DL, Drazen JM (2016) Data Sharing. N Engl J Med. 374(3):276-7. doi: 10.1056/NEJMe1516564
45. Gorgolewski KJ, Margulies DS, Milham MP (2013) Making data sharing count: a publication-based solution. Front Neurosci. 7:9. doi: 10.3389/fnins.2013.00009
46. Lo B, DeMets DL (2016) Incentives for clinical trialists to share data. N Engl J Med. 375 (12):1112-1115. doi: 10.1056/NEJMp1608351
47. Kaye J, Heeney C, Hawkins N, de Vries J, Boddington P (2009) Data sharing in genomics--re-shaping scientific practice. Nat Rev Genet. 10(5):331-5. doi: 10.1038/nrg2573
48. Groth P, Cousijn H, Clark T, Goble C (2020) FAIR data reuse – the path through data citation. Data Intelligence 2(1–2):78–86. doi: 10.1162/dint_a_00030
49. Robinson EL, Emanueli C, Martelli F, Devaux Y (2021). Leveraging non-coding RNAs to fight cardiovascular disease: the EU-CardioRNA network. European Heart Journal. 42(48), 4881–4883. doi: 10.1093/eurheartj/ehab326
50. Luo Y, Hitz BC, Gabdank I, Hilton JA, Kagda MS, Lam B, Myers Z, Sud P, Jou J, Lin K, Baymuradov UK, Graham K, Litton C, Miyasato SR, Strattan JS, Jolanki O, Lee JW, Tanaka FY, Adenekan P, O'Neill E, Cherry JM (2020) New developments on the Encyclopedia of DNA Elements (ENCODE) data portal. Nucleic Acids Res. 48(D1):D882-D889. doi: 10.1093/nar/gkz1062
51. Bandrowski A, Brinkman R, Brochhausen M, Brush MH, Bug B, Chibucos MC, Clancy K, Courtot M, Derom D, Dumontier M, Fan L, Fostel J, Fragoso G, Gibson F, Gonzalez-Beltran A, Haendel MA, He Y, Heiskanen M, Hernandez-Boussard T, Jensen M, Lin Y, Lister AL, Lord P, Malone J, Manduchi E, McGee M, Morrison N, Overton JA, Parkinson H, Peters B, Rocca-Serra P, Ruttenberg A, Sansone SA, Scheuermann RH, Schober D, Smith B, Soldatova LN, Stoeckert CJ Jr, Taylor CF, Torniai C, Turner JA, Vita R, Whetzel PL, Zheng J (2016) The Ontology for Biomedical Investigations. PLoS One 11(4):e0154556. doi: 10.1371/journal.pone.0154556
52. Prigent S, Valades-Cruz CA, Leconte L, Maury L, Salamero J, Kervrann C (2022) BioImageIT: Open-source framework for integration of image data management with analysis. Nat Methods. 19(11):1328-1330. doi: 10.1038/s41592-022-01642-9
53. Betsou F, Bulla A, Cho SY, Clements J, Chuaqui R, Coppola D, De Souza Y, De Wilde A, Grizzle W, Guadagni F, Gunter E, Heil S, Hodgkinson V, Kessler J, Kiehntopf M, Kim HS, Koppandi I, Shea K, Singh R, Sobel M, Somiari S, Spyropoulos D, Stone M, Tybring G, Valyi-Nagy K, Van den Eynden G, Wadhwa L (2016) Assays for Qualification and Quality Stratification of Clinical Biospecimens Used in Research: A Technical Report from the ISBER Biospecimen Science Working Group. Biopreserv Biobank. 14(5):398-409. doi: 10.1089/bio.2016.0018
54. Raynal B, Lenormand P, Baron B, Hoos S, England P. Quality assessment and optimization of purified protein samples: why and how? Microb Cell Fact. 2014 Dec 30;13:180. doi: 10.1186/s12934-014-0180-6
55. de Gonzalo-Calvo D, Sopić M, Devaux Y; EU-CardioRNA COST Action CA17129 (2022) Methodological considerations for circulating long noncoding RNA quantification. Trends Mol Med. 28(8):616-618. doi: 10.1016/j.molmed.2022.05.011
56. de Gonzalo-Calvo D, Pérez-Boza J, Curado J, Devaux Y; EU-CardioRNA COST Action CA17129 (2022) Challenges of microRNA-based biomarkers in clinical application for cardiovascular diseases. Clin Transl Med. 12(2):e585. doi: 10.1002/ctm2.585
57. de Gonzalo-Calvo D, Marchese M, Hellemans J, Betsou F, Skov Frisk NL, Dalgaard LT, Lakkisto P, Foy C, Scherer A, Garcia Bermejo ML, Devaux Y; EU-CardioRNA COST Action CA17129 (<https://cardiorna.eu/>) (2022) Consensus guidelines for the validation of qRT-PCR assays in clinical research by the CardioRNA consortium. Mol Ther Methods Clin Dev. 24:171-180. doi: 10.1016/j.omtm.2021.12.007
58. Vanhaverbeke M, Attard R, Bartekova M, Ben-Aicha S, Brandenburger T, de Gonzalo-Calvo D, Emanueli C, Farrugia R, Grillari J, Hackl M, Kalocayova B, Martelli F, Scholz M, Wettinger SB, Devaux Y; EU-CardioRNA COST Action CA17129 (2021) Peripheral blood RNA biomarkers for cardiovascular disease from bench to bedside: A Position Paper from the EU-CardioRNA COST Action CA17129. Cardiovasc Res. cvab327. doi: 10.1093/cvr/cvab327
59. Meinung B, Martin D, Zimmermann U (2019) Standardization in biobanking – between cooperation and competition. J Lab Med. 43(6):317-328. doi: 10.1515/labmed-2019-0105
60. Burton RAB, Rog-Zielinska EA, Corbett AD, Peyronnet R, Bodi I, Fink M, Sheldon J, Hoenger A, Calaghan SC, Bub G, Kohl P (2017) Caveolae in Rabbit Ventricular Myocytes: Distribution and Dynamic Diminution after Cell Isolation. Biophys J. 113(5):1047-1059. doi: 10.1016/j.bpj.2017.07.026
61. Lowndes JSS, Best BD, Scarborough C, Afflerbach JC, Frazier MR, O'Hara CC, Jiang N, Halpern BS (2017) Our path to better science in less time using open data science tools. Nat Ecol Evol. 1(6):160. doi: 10.1038/s41559-017-0160
62. Brazma A, Ball C, Bumgarner R, Furlanello C, Miller M, Quackenbush J, Reich M, Rustici G, Stoeckert C, Trutane SC, Taylor RC (2012). MINSEQE: Minimum Information about a high-throughput Nucleotide SeQuencing Experiment - a proposal for standards in functional genomic data reporting (1.0). Zenodo. doi: 10.5281/zenodo.5706412. Available at: https://zenodo.org/record/5706412
63. Füllgrabe A, George N, Green M, Nejad P, Aronow B, Fexova SK, Fischer C, Freeberg MA, Huerta L, Morrison N, Scheuermann RH, Taylor D, Vasilevsky N, Clarke L, Gehlenborg N, Kent J, Marioni J, Teichmann S, Brazma A, Papatheodorou I (2020) Guidelines for reporting single-cell RNA-seq experiments. Nat Biotechnol. 38(12):1384-1386. doi: 10.1038/s41587-020-00744-z
64. Skinnider, M.A., Squair, J.W. & Courtine, G (2021) Enabling reproducible re-analysis of single-cell data. Genome Biol 22, 215. doi: 10.1186/s13059-021-02422-y
65. Betsou F, Bilbao R, Case J, Chuaqui R, Clements JA, De Souza Y, De Wilde A, Geiger J, Grizzle W, Guadagni F, Gunter E, Heil S, Kiehntopf M, Koppandi I, Lehmann S, Linsen L, Mackenzie-Dodds J, Quesada RA, Tebbakha R, Selander T, Shea K, Sobel M, Somiari S, Spyropoulos D, Stone M, Tybring G, Valyi-Nagy K, Wadhwa L (2018) Standard PREanalytical Code Version 3.0. Biopreserv Biobank. 16(1):9-12. doi: 10.1089/bio.2017.0109
66. Percie du Sert N, Hurst V, Ahluwalia A, Alam S, Avey MT, Baker M, Browne WJ, Clark A, Cuthill IC, Dirnagl U, Emerson M, Garner P, Holgate ST, Howells DW, Karp NA, Lazic SE, Lidster K, MacCallum CJ, Macleod M, Pearl EJ, Petersen OH, Rawle F, Reynolds P, Rooney K, Sena ES, Silberberg SD, Steckler T, Würbel H (2020) The ARRIVE guidelines 2.0: updated guidelines for reporting animal research. BMJ Open Sci. 4(1):e100115. doi: 10.1136/bmjos-2020-100115
67. Alterovitz G, Dean D, Goble C, Crusoe MR, Soiland-Reyes S, Bell A, Hayes A, Suresh A, Purkayastha A, King CH, Taylor D, Johanson E, Thompson EE, Donaldson E, Morizono H, Tsang H, Vora JK, Goecks J, Yao J, Almeida JS, Keeney J, Addepalli K, Krampis K, Smith KM, Guo L, Walderhaug M, Schito M, Ezewudo M, Guimera N, Walsh P, Kahsay R, Gottipati S, Rodwell TC, Bloom T, Lai Y, Simonyan V, Mazumder R (2018) Enabling precision medicine via standard communication of HTS provenance, analysis, and results. PLoS Biol. 16(12):e3000099. doi: 10.1371/journal.pbio.3000099
68. Jacobsen JOB, Baudis M, Baynam GS, Beckmann JS, Beltran S, Buske OJ, Callahan TJ, Chute CG, Courtot M, Danis D, Elemento O, Essenwanger A, Freimuth RR, Gargano MA, Groza T, Hamosh A, Harris NL, Kaliyaperumal R, Lloyd KCK, Khalifa A, Krawitz PM, Köhler S, Laraway BJ, Lehväslaiho H, Matalonga L, McMurry JA, Metke-Jimenez A, Mungall CJ, Munoz-Torres MC, Ogishima S, Papakonstantinou A, Piscia D, Pontikos N, Queralt-Rosinach N, Roos M, Sass J, Schofield PN, Seelow D, Siapos A, Smedley D, Smith LD, Steinhaus R, Sundaramurthi JC, Swietlik EM, Thun S, Vasilevsky NA, Wagner AH, Warner JL, Weiland C; GAGH Phenopacket Modeling Consortium, Haendel MA, Robinson PN (2022) The GA4GH Phenopacket schema defines a computable representation of clinical data. Nat Biotechnol. 40(6):817-820. doi: 10.1038/s41587-022-01357-4
69. Kampmeijer R, Pavlova M, Tambor M, Golinowska S, Groot W (2016) The use of e-health and m-health tools in health promotion and primary prevention among older adults: a systematic literature review. BMC Health Serv Res. 16 (Suppl 5):290. doi: 10.1186/s12913-016-1522-3
70. Smith B, Magnani JW (2019) New technologies, new disparities: The intersection of electronic health and digital health literacy. Int J Cardiol. 292:280-282. doi: 10.1016/j.ijcard.2019.05.066
71. Linz D, Baumert M, Catcheside P, Floras J, Sanders P, Lévy P, Cowie MR, Doug McEvoy R (2018). Assessment and interpretation of sleep disordered breathing severity in cardiology: Clinical implications and perspectives. Int J Cardiol. 271:281-288. doi: 10.1016/j.ijcard.2018.04.076
72. van der Velden RMJ, Pluymaekers NAHA, Dudink EAMP, Luermans JGLM, Meeder JG, Heesen WF, Lenderink T, Widdershoven JWMG, Bucx JJJ, Rienstra M, Kamp O, van Opstal JM, Kirchhof CJHJ, van Dijk VF, Swart HP, Alings M, Van Gelder IC, Crijns HJGM, Linz D (2022) Mobile health adherence for the detection of recurrent recent-onset atrial fibrillation. Heart. 109(1):26-33. doi: 10.1136/heartjnl-2022-321346
73. Gawałko M, Hermans AN, van der Velden RM, Betz K, Vm Verhaert D, Hillmann HA, Scherr D, Meier J, Sultan A, Steven D, Terentieva E, Pisters R, Hemels M, Voorhout L, Lodziński P, Krzowski B, Gupta D, Kozhuharov N, Pison L, Gruwez H, Desteghe L, Heidbuchel H, Evens S, Svennberg E, de Potter T, Vernooy K, Pluymaekers NA, Manninger M, Duncker D, Sohaib A, Linz D, Hendriks JM (2022) Patient motivation and adherence to an on-demand app-based heart rate and rhythm monitoring for atrial fibrillation management: data from the TeleCheck-AF project. Eur J Cardiovasc Nurs. zvac061. doi: 10.1093/eurjcn/zvac061. Epub ahead of print.
74. Svennberg E, Tjong F, Goette A, Akoum N, Di Biase L, Bordachar P, Boriani G, Burri H, Conte G, Deharo JC, Deneke T, Drossart I, Duncker D, Han JK, Heidbuchel H, Jais P, de Oliviera Figueiredo MJ, Linz D, Lip GYH, Malaczynska-Rajpold K, Márquez M, Ploem C, Soejima K, Stiles MK, Wierda E, Vernooy K, Leclercq C, Meyer C, Pisani C, Pak HN, Gupta D, Pürerfellner H, Crijns HJGM, Chavez EA, Willems S, Waldmann V, Dekker L, Wan E, Kavoor P, Turagam MK, Sinner M (2022) How to use digital devices to detect and manage arrhythmias: an EHRA practical guide. Europace. 24(6):979-1005. doi: 10.1093/europace/euac038. Erratum in: Europace. 2022 Jul 15;24(6):1005
75. Grundy Q, Chiu K, Held F, Continella A, Bero L, Holz R (2019) Data sharing practices of medicines related apps and the mobile ecosystem: traffic, content, and network analysis. BMJ. 364:l920. doi: 10.1136/bmj.l920
76. Heijman J, Sutanto H, Crijns HJGM, Nattel S, Trayanova NA (2021) Computational models of atrial fibrillation: achievements, challenges, and perspectives for improving clinical care. Cardiovasc Res. 117(7):1682-1699. doi: 10.1093/cvr/cvab138
77. Clerx M, Beattie KA, Gavaghan DJ, Mirams GR (2019). Four Ways to Fit an Ion Channel Model. Biophys J. 117(12):2420-2437. doi: 10.1016/j.bpj.2019.08.001
78. Whittaker DG, Clerx M, Lei CL, Christini DJ, Mirams GR (2020) Calibration of ionic and cellular cardiac electrophysiology models. Wiley Interdiscip Rev Syst Biol Med. 12(4):e1482. doi: 10.1002/wsbm.1482
79. Clerx M, Cooling MT, Cooper J, Garny A, Moyle K, Nickerson DP, Nielsen PMF, Sorby H (2020) CellML 2.0. J Integr Bioinform. 17(2-3):20200021. doi: 10.1515/jib-2020-0021
80. The European Parliament and the Council of the European Union. General Data Protection Regulation (EU) 2016/679 (GDPR) <https://eur-lex.europa.eu/legal-content/EN/TXT/?uri=CELEX:02016R0679-20160504>. Accessed Jan 4, 2023
81. Bentzen HB, Høstmælingen N (2019) Balancing Protection and Free Movement of Personal Data: The New European Union General Data Protection Regulation. Ann Intern Med. 170(5):335-337. doi: 10.7326/M18-2782
82. Schwarz CG, Kremers WK, Therneau TM, Sharp RR, Gunter JL, Vemuri P, Arani A, Spychalla AJ, Kantarci K, Knopman DS, Petersen RC, Jack CR Jr (2019) Identification of Anonymous MRI Research Participants with Face-Recognition Software. N Engl J Med. 381(17):1684-1686. doi: 10.1056/NEJMc1908881
83. Adler ED, Voors AA, Klein L, Macheret F, Braun OO, Urey MA, Zhu W, Sama I, Tadel M, Campagnari C, Greenberg B, Yagil A (2020) Improving risk prediction in heart failure using machine learning. Eur J Heart Fail. 22(1):139-147. doi: 10.1002/ejhf.1628
84. Deist TM, Dankers FJWM, Ojha P, Scott Marshall M, Janssen T, Faivre-Finn C, Masciocchi C, Valentini V, Wang J, Chen J, Zhang Z, Spezi E, Button M, Jan Nuyttens J, Vernhout R, van Soest J, Jochems A, Monshouwer R, Bussink J, Price G, Lambin P, Dekker A (2020) Distributed learning on 20 000+ lung cancer patients - The Personal Health Train. Radiother Oncol. 144:189-200. doi: 10.1016/j.radonc.2019.11.019
85. Lenz S, Hess M, Binder H (2021) Deep generative models in DataSHIELD. BMC Med Res Methodol. 21(1):64. doi: 10.1186/s12874-021-01237-6
86. Becker M (2022) Swarm learning for decentralized healthcare. Hautarzt. 73(4):323-325. doi: 10.1007/s00105-021-04940-z
87. Griebel L, Hinderer M, Amr A, Meder B, Schweig M, Deuber D, Egger C, Kawohl C, Krämer A, Flade I, Schröder D, Prokosch HU (2020) The Patient as Genomic Data Manager – Evaluation of the PROMISE App. Stud Health Technol Inform. 270:1061-1065. doi: 10.3233/SHTI200324
88. Seltzer E, Goldshear J, Guntuku SC, Grande D, Asch DA, Klinger EV, Merchant RM (2019) Patients' willingness to share digital health and non-health data for research: a cross-sectional study. BMC Med Inform Decis Mak. 19(1):157. doi: 10.1186/s12911-019-0886-9
89. Mello MM, Lieou V, Goodman SN (2018) Clinical Trial Participants' Views of the Risks and Benefits of Data Sharing. N Engl J Med. 378(23):2202-2211. doi: 10.1056/NEJMsa1713258
90. Zenker S, Strech D, Ihrig K, Jahns R, Müller G, Schickhardt C, Schmidt G, Speer R, Winkler E, von Kielmansegg SG, Drepper J (2022) Data protection-compliant broad consent for secondary use of health care data and human biosamples for (bio)medical research: Towards a new German national standard. J Biomed Inform. 131:104096. doi: 10.1016/j.jbi.2022.104096
91. Deutsche Stiftung Organstransplantation (2022) Jahresbericht 2021 Organspende und Transplantation in Deutschland. Deutsche Stiftung Organtransplantation, Frankfurt am Main. Available at: <https://www.dso.de/SiteCollectionDocuments/DSO-Jahresbericht%202021.pdf>. Accessed Dec 21, 2022
92. European Commission, Directorate-General for Research and Innovation (2019) Cost-benefit analysis for FAIR research data: cost of not having FAIR research data. Publications Office, Brussels, Belgium. <https://data.europa.eu/doi/10.2777/02999>. Accessed Jan 4, 2023
93. Chue Hong NP, Katz DS, Barker M, Lamprecht A-L, Martinez C, Psomopoulos FE, Harrow J, Castro LJ, Gruenpeter M, Martinez PA, Honeyman T, Struck A, Lee A, Loewe A, van Werkhoven B, Jones C, Garijo D, Plomp E, Genova F, Shanahan H, Leng J, Hellström M, Sandström M, Sinha M, Kuzak M, Herterich P, Zhang Q, Islam S, Sansone S-A, Pollard T, Atmojo UD, Williams A, Czerniak A, Niehues A, Fouilloux AC, Desinghu B, Goble C, Richard C, Gray C, Erdmann C, Nüst D, Tartarini D, Ranguelova E, Anzt H, Todorov I, McNally J, Moldon J, Burnett J, Garrido-Sánchez J, Belhajjame K, Sesink L, Hwang L, Tovani-Palone MR, Wilkinson MD, Servillat M, Liffers M, Fox M, Miljković N, Lynch N, Martinez Lavanchy P, Gesing S, Stevens S, Martinez Cuesta S, Peroni S, Soiland-Reyes S, Bakker T, Rabemanantsoa T, Sochat V, Yehudi Y, RDA FAIR4RS WG (2022) FAIR Principles for Research Software (FAIR4RS Principles) (1.0). Zenodo. doi: 10.15497/RDA00068. Available at: <https://zenodo.org/record/6623556#.YqCJTJNBwlw>
94. Gibson F, Overton P, Smulders T, Schultz S, Eglen S, Ingram C, Panzeri S, Bream P, Whittington M, Sernagor E, Cunningham M, Adams C, Echtermeyer C, Simonotto J, Kaiser M, Swan D, Fletcher M, Lord P (2008) Minimum Information about a Neuroscience Investigation (MINI) Electrophysiology. Nat Prec. doi: 10.1038/npre.2008.1720.1
95. Quinn TA, Granite S, Allessie MA, Antzelevitch C, Bollensdorff C, Bub G, Burton RA, Cerbai E, Chen PS, Delmar M, Difrancesco D, Earm YE, Efimov IR, Egger M, Entcheva E, Fink M, Fischmeister R, Franz MR, Garny A, Giles WR, Hannes T, Harding SE, Hunter PJ, Iribe G, Jalife J, Johnson CR, Kass RS, Kodama I, Koren G, Lord P, Markhasin VS, Matsuoka S, McCulloch AD, Mirams GR, Morley GE, Nattel S, Noble D, Olesen SP, Panfilov AV, Trayanova NA, Ravens U, Richard S, Rosenbaum DS, Rudy Y, Sachs F, Sachse FB, Saint DA, Schotten U, Solovyova O, Taggart P, Tung L, Varró A, Volders PG, Wang K, Weiss JN, Wettwer E, White E, Wilders R, Winslow RL, Kohl P (2011) Minimum Information about a Cardiac Electrophysiology Experiment (MICEE): standardised reporting for model reproducibility, interoperability, and data sharing. Prog Biophys Mol Biol. 107(1):4-10. doi: 10.1016/j.pbiomolbio.2011.07.001
96. Rocca-Serra P, Gu W, Ioannidis V, Daloii TA, Capella-Gutierrez S, Chandramouliswaran I, Splendiani A, Burdett T, Giessmann RT, Henderson D, Batista D, Lister A, Emam I, Gadiya Y, Giovanni L, Willighagen E, Evelo C, Gray AJG, Gribbon P, Juty N, Welter D, Quast K, Peeters P, van Vlijmen H, Plasterer T, Reilly D, van der Horst E, Sansone SA, the FAIR Cookbook Recipes' Authors (2022) The FAIR Cookbook - the essential resource for and by FAIR doers. Zenodo. doi: 10.5281/zenodo.7156792. Available at <https://zenodo.org/record/7156792>
97. Hrynaszkiewicz, I., Simons, N., Hussain, A., Grant, R. and Goudie, S. (2020) Developing a Research Data Policy Framework for All Journals and Publishers. Data Science Journal. 19(1):5. doi: 10.5334/dsj-2020-005

**Tables and Figures**

**Table 1.** Examples of standards and tools that researchers may use to make their data FAIR.

| **Example** | **Description** |
| --- | --- |
| Research Data Alliance (RDA)  <https://www.rd-alliance.org/> | A global, community-driven initiative to build social and technical infrastructure for open sharing and reuse of data, with several working groups in a number of disciplines |
| FAIRsharing  [www.fairsharing.org](http://www.fairsharing.org/) | A searchable, interconnected registry of data standards, databases, and data policies across many research areas, allowing researchers to discover relevant repositories that meet their requirements [24] |
| The FAIR Cookbook  <https://faircookbook.elixir-europe.org> | A collection of practical ‘recipes’ that provide guidance on the operational steps of FAIR data management, from creating unique, persistent identifiers to declaring data’s permitted uses [96] |
| FAIRassist.org  <https://fairassist.org> | A repository aiming to offer personalised guidance to discover FAIR standards and other resources such as the Data Stewardship Wizard |
| FAIR Data Self Assessment Tool  <https://ardc.edu.au/resource/fair-data-self-assessment-tool/> | Self-assessment tool from the Australian Research Data Commons that allows users to assess how FAIR their research dataset is by answering simple questions |
| ELIXIR Research Data Management Kit (RDMKit)  <https://rdmkit.elixir-europe.org/> | Provides a set of best practices and guidelines for FAIR RDM across several life science domains, and journal research data policies [97] |
| OpenAIRE  <https://www.openaire.eu> | Provides resources for researchers for the management and interoperability of data |
| Minimum Information about a Cardiac Electrophysiology Experiment (MICEE) [95] | An example of minimum reporting standards for recording, annotating, and reporting data from cardiac electrophysiology experiments |
| ‍FAIRsFAIR Data Policy Checklist [https://www.fairsfair.eu/sites/default/files/ FsF_Structured_Policy_Descriptions_ 17022022.pptx.pdf](https://www.fairsfair.eu/sites/default/files/FsF_Structured_Policy_Descriptions_17022022.pptx.pdf) | The FAIRsFAIR FAIR Data Policy Checklist and related structured policy description template provide support for the creation of structured policy documents at the project, institutional, and community level, helping policy-makers to assess whether elements of their data policies are FAIR-enabling |

**Figure 1.** The data and metadata lifecycle.

PID, permanent identifier; QC, quality control.
